# Significance of mechanical loading in bone fracture healing, bone regeneration, and vascularization

**DOI:** 10.1177/20417314231172573

**Published:** 2023-05-22

**Authors:** Qianli Ma, Zahra Miri, Håvard Jostein Haugen, Amirhossein Moghanian, Dagnjia Loca

**Affiliations:** 1 Department of Biomaterials, Institute of Clinical Dentistry, University of Oslo, Norway; 2 Department of Immunology, School of Basic Medicine, Fourth Military Medical University, Xi’an, PR China; 3 Department of Materials Engineering, Isfahan University of Technology, Isfahan, Iran; 4 Department of Materials Engineering, Imam Khomeini International University, Qazvin, Iran; 5 Rudolfs Cimdins Riga Biomaterials Innovations and Development Centre, Institute of General Chemical Engineering, Faculty of Materials Science and Applied Chemistry, Riga Technical University, Riga, Latvia; 6 Baltic Biomaterials Centre of Excellence, Headquarters at Riga Technical University, Riga, Latvia

**Keywords:** Bone, mechanical loading, remodeling, fracture healing, vascularization, bone regeneration, bone structure

## Abstract

In 1892, J.L. Wolff proposed that bone could respond to mechanical and biophysical stimuli as a dynamic organ. This theory presents a unique opportunity for investigations on bone and its potential to aid in tissue repair. Routine activities such as exercise or machinery application can exert mechanical loads on bone. Previous research has demonstrated that mechanical loading can affect the differentiation and development of mesenchymal tissue. However, the extent to which mechanical stimulation can help repair or generate bone tissue and the related mechanisms remain unclear. Four key cell types in bone tissue, including osteoblasts, osteoclasts, bone lining cells, and osteocytes, play critical roles in responding to mechanical stimuli, while other cell lineages such as myocytes, platelets, fibroblasts, endothelial cells, and chondrocytes also exhibit mechanosensitivity. Mechanical loading can regulate the biological functions of bone tissue through the mechanosensor of bone cells intraosseously, making it a potential target for fracture healing and bone regeneration. This review aims to clarify these issues and explain bone remodeling, structure dynamics, and mechano-transduction processes in response to mechanical loading. Loading of different magnitudes, frequencies, and types, such as dynamic versus static loads, are analyzed to determine the effects of mechanical stimulation on bone tissue structure and cellular function. Finally, the importance of vascularization in nutrient supply for bone healing and regeneration was further discussed.

## Introduction

Bone is a mineralized connective tissue that provides a structural framework for vertebrates, serving functions such as locomotion, support and protection of soft tissues, minerals storage, and hemopoiesis.^1,2^ As a metabolically active organ, bone undergoes continuous remodeling, repair, and regeneration throughout life. After a fracture, the formation of new bone involves the interaction between molecules and different cell lineages.^3^ The ultimate goal of healing is to improve the load-bearing ability and restore bone strength,^4^ which may be affected by factors such as unhealthy habits like smoking, lack of nutrients, biological factors like growth hormones and cytokines, and genetic factors, and physical stimuli like ultrasound, mechanics, and electrical fields.^4^ Among the abovementioned factors, mechanical loading has gathered extensive attention as a potential therapeutic strategy for promoting bone regeneration, owing to its ubiquitous nature, non-invasiveness, and maneuverability. The impact of mechanical loads on bone regeneration has been widely studied since Wolff discovered that mechanical loads could promote bone regeneration.^5^ Another well-known “Mechanostat” hypothesis invented by Harold Frost in 1960 described how mechanical loading influences bone structure by changing the bone mass and architecture to provide a design that resists habitual loads with optimal use of material accordingly.^6–8^ Mechanical forces can stimulate bone marrow mesenchymal cell congregation in the initial fracture healing phase, promote callus tissue formation during the repairing phase, and improve tissue reconstruction in the remodeling phase.^9^ The osteoblastic cells are also sensitive to mechanical loading and respond to it by altered proliferation, extracellular matrix synthesis, and secretion/expression of cytokines.^10^

However, the outcome of bone remodeling relies on the balance of osteogenic and osteoclastic activity. The effects of mechanical loading on osteoblastic cells depend on the type and magnitude of the stimulation. Inappropriate stimulation can hinder osteogenic functions while promoting the overactivation of osteoclasts.^11^ Furthermore, osteoblasts from patients with osteoporosis failed to increase their proliferation and TGF-β release in response to a mechanical loading regimen that stimulated normal donor osteoblasts, suggesting that the response mode of bone formation-related cells to mechanical loading is not fixed but highly correlated with the overall health condition of the host such as age, sex, disease, etc.^12^ It also indicates that mechanical loading, as an initiating factor of bone remodeling, cannot function independently regardless of bone tissue’s biophysical and biochemical microenvironment. Instead, such regulation is more likely achieved through the modulation of mechanotransduction signaling pathways, the interaction between physiological, biochemical, and mechanobiological signals, and the local cytokine profile.^13,14^ In addition to its effects on bone remodeling, mechanical loading can also affect bone vascularization, which is critical for nutrient supply, waste exchange, and the long-term stability of bone.^15^

This work aims to comprehensively review current studies on regulating bone healing and regeneration through mechanical loading. Regarding previous studies based on different mechanical models, a consensus on the biological functions of mechanical stimuli and relevant mechanisms has not been reached. To address this issue, bone structure, bone cells, the processes involved in bone remodeling, mechanoconduction, and responses to mechanical loading were defined and explained first. Then, the effects and related mechanisms of mechanical loading on key events such as fracture healing and vascularization in vitro/in vivo were then systematically analyzed. Finally, potential therapeutic strategies and future research directions for improving bone healing/regeneration by optimizing the parameters of mechanical stimulation were further discussed.

## Biology and mechanosensation of bone matrix and bone cells

**Figure 1.**
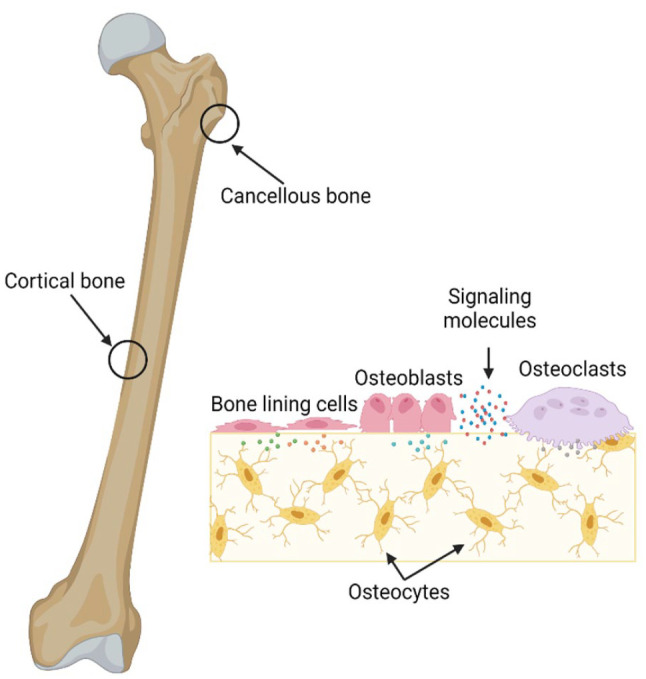
Bone includes both cortical and trabecular bone. In the cellular area, bone comprises osteoblasts, osteocytes, bone lining cells, and osteoclasts created by BioRender 2023.

There are two types of bone: dense cortical bone and spongy cancellous bone (Figure 1). Cortical bone forms a dense protective shell around the medullary canal and stores yellow marrow. The osteons within the dense cortical bone are arranged in concentric rings called lamellae, which contain cells crucial for bone formation and remodeling. The Haversian canal, located in the center of each osteon, houses blood vessels, lymphatic vessels, and nerve fibers.^16^ Cancellous bone comprises a cellular network of trabeculae grouped in arrangements that follow the lines of stress points, allowing maximum strength with minimal mass. Red bone marrow is located between each trabecular pore and contains hematopoietic stem cells, which play a critical role in hematopoiesis.^16^ Bone is composed of 60% inorganic minerals, 30% organic components, and 10% water.^17,18^ As a vital and uniquely biodynamic organ, bone contains a matrix supporting its spatial structure and bone cells. The bone matrix consists of organic components and inorganic minerals.^17^ The organic matrix contains ~90% collagens (mainly type I collagen) and non-collagenous proteins including osteocalcin (OCN), bone sialoprotein (BSP), osteopontin (OPN), and bone morphogenetic proteins (BMPs), etc.^19^ While phosphate and calcium ions comprise the primary inorganic substance of bone, other minerals such as bicarbonate, sodium, potassium, citrate, magnesium, carbonate, fluorite, zinc, barium, and strontium are also present in significant amounts and contribute to the structure and strength of bone.^20,21^ Hydroxyapatite (HA) is the main inorganic crystal in bone tissue, forming through the nucleation of calcium and phosphate. Collagen and non-collagenous matrix proteins work together to create a cross-linked framework for HA deposition and matrix mineralization, forming the structural basis for bone tissue’s characteristic stiffness and resistance.^2^ Alongside the supporting functions of bone strength and homeostasis, the bone matrix provides several soluble or adhesion molecules that regulate the bioactivity of bone cells, thereby participating in bone remodeling and metabolism.^22^ Moreover, depending on the arrangement of the hierarchical structural units, the mineralized bone matrix can decompose and transform the mechanical loadings into cells in the form of compressive stress, tensile strain, or fluid shear stress (FSS).^23^

Four key cell types are responsible for bone’s dynamic development, remodeling, and healing: osteoblasts, osteoclasts, bone lining cells, and osteocytes.

(1) Osteoblasts, known for their bone formation functions, locate along the bone surface, secrete osteoid toward the bone matrix and make up around 4%–6% of the total resident bone cells.^24,25^ These cuboidal cells (diameter of 9.33–29.91 μm) have abundant rough endoplasmic reticulum, a prominent Golgi apparatus, and various secretory vesicles, indicating their role in protein synthesis in the bone matrix.^26,27^ Osteoblasts are derived from mesenchymal stem cells (MSCs) under the conservative timely programmed steps (MSCs-osteoblast progenitors-osteoblasts), such as BMPs synthesis, Wnt pathways activation, and upregulated expression of *Runx2.*^28–30^ The maturation of osteoblasts is characterized by increased secretion of collagen I and non-collagen bone matrix proteins, such as OPN, OCN, and BSP.^30–32^ Osteoblasts synthesize bone matrix in two stages: osteoblasts secrete collagens, non-collagenous proteins, and proteoglycans, including decorin and biglycan in the first stage. Then, the bone matrix undergoes mineralization in two phases: the vesicular and fibrillar phases.^33,34^ In the vesicular phase, matrix vesicles (30–200 nm in diameter) are released from the apical membrane domain of the osteoblasts into the newly formed bone matrix, where they bind to proteoglycans and other organic components.^35^ The negatively charged sulfated proteoglycans immobilize calcium ions within the vesicles.^34^ When proteoglycans are degraded by enzymes produced by osteoblasts, the calcium ions are released and enter into vesicles through the annexin-associated calcium channels.^33^ Concurrently, ALP secreted by osteoblasts degrades phosphate-containing compounds, releasing phosphate ions into the matrix vesicles. The phosphate and calcium ions inside the vesicles nucleate and form HA crystals.^36^ During the fibrillar phase, matrix vesicles rupture because of the supersaturation of calcium and phosphate ions inside, allowing the HA crystals to spread to the surrounding matrix.^37^ At the end of the bone-forming phase and with the maturation of the bone matrix, osteoblasts will enter into three different fates: (i) embedded in the bone matrix and differentiate into osteocytes, (ii) transform into quiescent flat-shaped bone lining cells that cover the bone surfaces, (iii) undergo apoptosis.^38^ As the main contributor to bone formation, osteoblasts have been proven to respond to mechanical stimuli. Hyper gravity at 3×*g* could upregulate the osteogenic mRNA expression, including *ALP, Runx2, OPN, OCN, and Osterix* of osteoblasts.^39,40^ Meanwhile, microgravity inhibits the osteoprotegerin (OPG, a potent decoy receptor/inhibitor of receptor activator of nuclear factor kappa-B ligand, RANKL) production from osteoblasts and leads to high RANKL/OPG ratio and increased osteoclasts formation.^41^ Their osteogenic functions could also be promoted under the stimulation of tensile strain.^42^

(2) Bone lining cells are quiescent osteoblasts with a flat shape and a diameter of around 15 μm. They cover the bone surface and inhibit bone resorption by preventing the direct interaction between bone matrix and osteoclasts.^43^ There is a layer of unmineralized osteoid between bone lining cells and mineralized bone. With various surface receptors, bone lining cells could respond to signaling molecules (e.g. Parathyroid Hormone-PTH, prostaglandin E_2_ - PGE_2_) by removing the unmineralized covering osteoid, thereby exposing the mineralized underlying bone matrix to osteoclasts and initiating the bone resorption.^44^ By anchoring hematopoietic stem cells, bone lining cells also provide appropriate signals to keep these stem cells in an undifferentiated state.^45^ On the other hand, bone lining cells play a crucial role in the transitions involved with bone remodeling by communicating through gap junctions with osteocytes deep inside the bone matrix. They also participate in the formation of osteoclasts by producing RANKL and OPG.^43,45^ Although bone lining cells do not synthesize new bone, they regulate osteoblastic and osteoclastic activity and mechanosensation.^46^ They may change back to an osteoblastic phenotype in the presence of parathyroid hormone or specific physiological status of bone.^47,48^

(3) Osteocytes are the most abundant and long-lived bone cells (up to 25 years lifespan), making up 90%–95% of the total bone cells. They are derived from the MSCs lineage through osteoblastic differentiation and undergo four identifiable stages: osteoid-osteocytes, pre-osteocytes, young osteocytes, and mature osteocytes.^38^ During the osteoblasts-osteocytes transition, cytoplasmic processes begin to appear, followed by progressive encapsulation of osteocytes into the bone matrix.^26^ Morphological and ultrastructural changes occur during this process, such as a reduced size of rounded osteocytes, a decreased number of organelles (e.g. Golgi apparatus), and an increase in the ratio of nucleus-to-cytoplasm, reflecting a decline in protein synthesis and secretion. When mature osteocytes are fully embedded in the mineralized bone matrix, the expression of osteoblast-specific markers is downregulated. In contrast, osteocytic markers such as dentine matrix protein 1 (DMP1) and sclerostin (Sost) are highly expressed.^49,50^ Osteocytes display a dendritic morphology in the lacunae (a typical dimension of 9–29 μm in length and 2–8 μm in width), which are wrapped by the mineralized bone matrix (Figure 1). Their cytoplasmic processes cross tiny tubes named canaliculi (with a diameter of 100–700 nm), forming the osteocyte lacunar-canalicular system.^51,52^ These processes are connected by gap junctions to adjacent osteocytic processes, the cytoplasmic protrusions of osteoblasts, and bone lining cells on the bone surface, allowing the intercellular exchange of small molecules such as NO.^53^ Intercellular communication is also achieved by interstitial fluid flowing between the osteocytes processes and canaliculi (50–100 nm channel size).^54^ The osteocytes function as mechanosensor through the lacunar-canalicular system, as their interconnected network can detect mechanical loading, aiding in the adaptation of bone to daily mechanical forces.^55^ The morphology of embedded osteocytes varies by bone type. Osteocytes from trabecular bone are more rounded than elongated cortical bone osteocytes.^56^ Such difference is not only affected by the arrangement of the basic unit of bone substance but also more likely to be the differential feedback of osteocytes to the stress distribution in different bone types. By altering the synthesis of various signaling molecules such as BMPs, Wnts, PGE2, and NO, osteocytes orchestrate bone remodeling by manipulating the differentiation, activation, and recruitment of osteoblasts and osteoclasts in response to mechanical stimuli.^57–61^ Experimental evidence from Xiong et al. and Nakashima et al. indicated that RANKL deletion in osteocytes leads to resistance to bone loss induced by mechanical unloading and osteopetrosis phenotype in mouse model.^62,63^ Tatsumi et al. reported that mice exhibited fragile bone with intracortical porosity, microfractures, and other hallmarks in aging bone tissue after selective ablation of osteocytes. these osteocytes-defect mice were highly resistant to the mechanical unloading-induced bone loss, which directly support the role of osteocytes in mechanotransduction.^64^ As a source of OPG, osteocytes regulate osteoclast-mediated bone resorption through differential secretion profiles of OPG and RANKL.^65^ Additionally, osteocytes also respond to fluid pressure. Kulkarni et al. established an in vitro model to study the remodeling capacity of osteocytes under pulsating fluid flow (PFF), which ubiquitously exists in bone matrix. PPF (0.7 ± 0.3 Pa, 5 Hz) application upregulated OPG expression via a matrix extracellular phosphoglycoprotein (MEPE)-related manner, thereby inhibiting mouse osteoclasts formation dramatically.^66^

4) Osteoclasts are terminally differentiated cells. They originate from monocytic cells of the hematopoietic stem cell lineage in the bone marrow and appear as large (varying diameter: 10–300 μm) multinucleated cells. The development of osteoclasts is influenced by several factors, such as macrophage colony-stimulating factor (M-CSF) and RANKL secreted from osteoblastic lineage cells and stromal cells.^67,68^ As the main cellular component mediating bone resorption (Figure 1), osteoclasts release protons and proteases (cathepsin, tartrate-resistant acid phosphatase (TRAP), and matrix metalloproteinases (MMPs) et al.) to create an acidic environment conducive to mineral dissolution and bone matrix proteins’ degradation.^69–71^ In addition to their well-studied osteolytic functions, osteoclasts were also reported as mechanosensitive cells. The fluid surrounding the osteoclasts in the lacunar-canalicular matrix enables the exchange of metabolic and biochemical signaling molecules and generates flow-based mechanical stimuli throughout skeletal loading.^24^ Therefore, current studies on the response of osteoclasts to mechanical stimuli were mainly conducted through hydrodynamic models. FSS has been reported to alter the cell shape and *ATP6V1A* and *TCIRG1* expression in rat osteoclasts without affecting cell viability.^72^ FSS could further inhibit the osteoclasts’ differentiation and bone resorption functions of mature osteoclasts via the ERK5 pathway.^73^ Another possible explanation is that FSS mediates the influx of calcium ions through STIM and transient receptor potential vanilloid 4 (TRPV4) Ca^2+^ channels on osteoclast progenitors at the early and late stages of osteoclast differentiation separately, thereby affecting osteoclast formation.^74^ The application of FSS does not always inhibit the differentiation and function of osteoclasts but depends on the magnitude and duration of FSS per cycle.^75^ Physiological FSS loading (loading amplitude of 0.7 ± 0.3 Pa) could inhibit osteoclast differentiation from hematopoietic progenitor cells, whereas higher FSS loading (loading amplitude of 3.0 ± 0.2 Pa) dramatically increased osteoclast formation. Similarly, under higher FSS load, longer loading duration per cycle resulted in more osteoclasts formation, ATP release, and bone resorption areas.^75^ In addition to hydrodynamic models, tension stimulation (stretching model) was also reported to affect osteoclast differentiation. Different studies have produced inconsistent findings, indicating that the intensity of mechanical strain, loading frequency and duration of loading application per cycle, and the total duration of force application utilized in various models may play a crucial role in the differentiation and functionalization of osteoclasts.^76,77^

## Bone remodeling, mechanosensation, and mechanotransduction under mechanical loadings

### Bone remodeling in response to mechanical loadings

Bone remodeling is a continuous and dynamic process to resorb and replace tiny tissue packets involving the coordination of osteogenic and osteolytic activities, which is achieved by the concerted functions of osteoblasts, osteocytes, bone lining cells, and osteoclasts cells in anatomical structures named “basic multicellular units” (BMU, also known as bone remodeling units, BRU). Bone remodeling occurs on all kinds of bone surfaces, including the bone on the periosteal and endosteal sides, Haversian canals, and the surface of the trabecular bone. Etc. Trabecular bone has a dramatically higher remodeling rate (5–10 times) than cortical bone in adults. The rate of cortical bone remodeling may be up to 50% per year in the first 2 years of life and then reduce to 2%–5% per year in older individuals.^78^ Various factors, including system health conditions, age, hormone level, cytokine profiles, and mechanical loading, tightly regulate the activities of bone cells and ultimately decide the fate of bone remodeling. The total bone quantity will decrease if bone resorption exceeds bone formation over the years.

**Figure 2.**
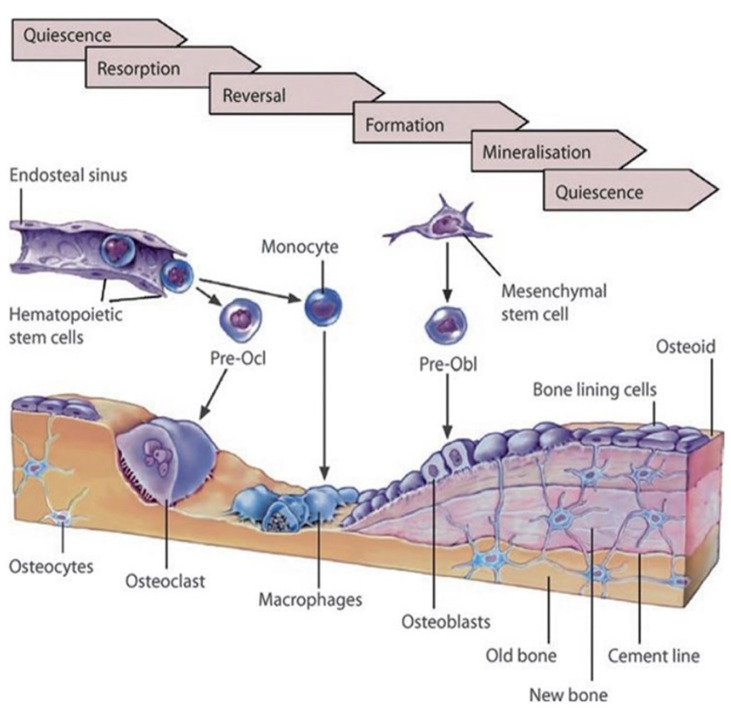
The schematic of the bone remodeling process^85^ was adapted from Reiner and Christoph and reprinted with permission from © 2020 Springer Nature Switzerland AG by license number 501718237.

Three possible explanations have been suggested for this negative skewing of bone metabolism: (i) enhanced osteoclastic activity without enhanced osteoblastic activity (high turnover), (ii) regular osteoclastic activity but with decreased osteoblastic activity (low turnover), and (iii) decreased osteoclastic activity with decreased osteoblastic activity (atrophic or adynamic bone). The decrease in bone quantity primarily attributes to the lack of coordination between BMUs, which comprise the cutting cone formed first by osteoclasts and the closing cone formed subsequently by osteoblasts (Figure 2) accompanied by the participation of blood vessels and the peripheral innervation.^79^ Both loss and bone gain result from skewed bone remodeling. Anabolic remodeling can increase net bone mass in response to more significant physical activity. For example, the playing arms (humeri) of professional tennis players have 20% more bone mineral mass than the non-playing arms, mainly due to increased diaphyseal thickness.^80,81^ In contrast, bone loss is associated with prolonged bed rest, spinal cord injury, or space travel.^82–84^ A typical remodeling process takes about 120 days and is divided into 6 steps/phases.^85^

(i) Quiescence phase: a layer of bone lining cells over a thin collagenous membrane covers the bone surface.
(ii) Activation phase: the quiescent surface of the bone is prepared for resorption, which involves the retraction of the bone lining cells and the elimination of the collagenous membrane covering the bone’s surface. MMPs produced by osteoblasts are involved in this process. The site-specific activation might be obtained by the mechanical stresses, which are transmitted to the endosteal lining cells by the osteocytes via the lacunar-canalicular network.
(iii) Resorption phase: osteoclastic precursors (e.g. monocytes, macrophages, multinuclear giant cells, etc.). Osteoclasts develop ruffled membranes, form cutting cones, and resorb the bone, forming lacunae or pits. Meanwhile, osteoclasts immigrate slowly or undergo apoptosis.
(iv) Reversal phase: Osteoblast progenitors are driven to the resorption pit. At the same time, macrophages prepare the surface of the resorption pit for new bone formation by eliminating the debris of osteoclasts.
(v) Early and late formation phase: active osteoblasts produce osteoid, followed by osteoid mineralization.
(vi) Quiescence phase: finally, the osteoblasts undergo apoptosis or differentiate into flat bone lining cells or osteocytes if trapped inside the newly formed bone matrix.^85^

Mechanical loading-mediated bone remodeling is not an independent process involving a single factor. More and more investigations have been focused on the cytokines, genes encoding the enzymes, bone matrix proteins, and transcription factors regulating local bone remodeling. Besides, exercise-generated loads can regulate the level of PTH, estrogen, and glucocorticoids, which mediates cytokines production and skews the anabolic/catabolic balance of bone remodeling at the system level.^86^ For example, estrogen can prevent bone resorption by inhibiting RANKL secretion and TRPV5 (a non-selective Ca^2+^ ion channel) expression while promoting osteoblastic OPG production.^87,88^ Physical exercises inhibit the secretion of proinflammatory cytokines (such as IL-1, IL-6, and TNF-α. etc.), facilitating bone resorption while stimulating the protective cytokines production against bone resorption (such as IL-10, IL-2, IL-12, and IL-4) by OPG/RANKL/RANK-independent pathways.^89–91^ It was reported that the osteocytes and osteoblasts in the bone could respond to both fluid flow and mechanical deformation, which result from mechanical loading in vivo.^92^ Famous mediators of mechanical loading-induced bone formation include NO, PGE_2_, prostaglandin I2 (PGI2), and glucose-6-phosphate dehydrogenase (G6PD).^93^ In vitro investigations on osteoblasts and osteocytes have demonstrated that the level of prostanoids and NO increased after exposure to physiological fluid flow and mechanical strain.^94^ Mechanical stimuli acting on bone marrow stromal cells could suppress RANKL expression and osteoclast formation. Osteoblastic lineage cells are likely to inhibit bone resorption via NO production.^61^ Two active prostaglandins, PGI2 and PGE_2_, are released from osteocytes or osteoblasts shortly after mechanical loading and mediate the recruitment of osteoblasts from bone marrow.^95,96^ Subcutaneous administration of PGE_2_ in canines considerably enhances bone formation on periosteal and endocortical surfaces, with apparent trabecular bone formation inside bone marrow.^97^

Only a few interventional strategies have been proposed to address the problems associated with adverse bone remodeling. One strategy is avoiding bone resorption or improving osteoblast activity, which can be achieved by manipulating bone remodeling through biochemical mediators or hormones (estrogens and anticatabolic drugs, such as calcitonin, bisphosphonates, and selective estrogen receptor modulators (SERMs)).^98^ Nevertheless, these strategies fail to utilize the intrinsic ability of bone tissue to adapt and respond to external loading, which is based on the natural and appropriate coordination between osteolysis and osteogenesis at specific bone sites under mechanical loadings. However, at the cellular level, osteoblasts, osteoclasts, and osteocytes have different mechano-sensitivity. Different research models of mechanical loadings (loading types, frequency, magnitude, etc.) also lead to controversial impacts on bone remodeling. Therefore, the structural basis and mechanisms of mechanosensation and mechanotransduction in bone tissue need to be discussed detailedly.

### Mechanosensation and mechanotransduction: Structures and mechanisms

With typical macro-micron-nano hierarchical architectural structures, bone matrix can transmit and transform mechanical loadings to bone cells in forms of compressive stress, tensile strain, FSS, etc.^23^ Bone tissue deformation during everyday locomotion ranges from 0.04% to 0.3%, with a rare occurrence exceeding 0.1%.^99^ In vitro studies reveal that the deformation required for bone cells to react to mechanical stimulation is significantly higher, ranging from 1% to 10%, which is 10 to 100 times greater than that needed for bone tissue. It is important to note that using the same relative deformation to stimulate bone cells in natural bone tissue would result in a fracture.^100,101^ You et al.’s experimental mathematical model explains the contradiction between macroscopic and microscopic stimulation levels. The model suggests that the canalicular system, where osteocytes are embedded, acts as an amplifier for the mechanical deformation generated by physical activity. According to Weinbaum’s model, mechanical loading-mediated fluid flow goes through the canalicular space. It deforms the shape of tethering elements (dendritic processes of osteocytes are tethered to the canalicular wall and anchored to hexagonal actin bundles within the cell processes), generating a drag force that then applies a hoop strain on the central actin bundles inside the cell processes of osteocytes.^102^ This system allows for more significant deformation at the cellular level than using the same level of deformation at the macroscopic level.^101^ In addition, FSS is also significant in affecting the bone matrix components and tailors the functions of bone cells in vivo by acting on the endosteal bone surfaces, walls of lacunar-canalicular system, cell membranes as well as collagens in as-formed osteoid.^23,103–105^ A recent study revealed that osteocytes do not always connect permanently with the bone surface cells but with highly dynamic structures.^106^ Mechanical loading-mediated fluid flow exerts FSS on osteocytes, resulting in the deformation of cells and dendric processes within the lacunar-canalicular system.^107^ The theoretical model predicts that peak physiological loadings will effectively make wall shear stress on osteocytes in vivo from 0.8 to 3.0 Pa (8–30 dyn/cm^2^).^107^

Three levels of porosity in the bone matrix are hierarchically nested within microcirculatory pathways and contribute to the generation of fluid flow under mechanical loadings.^108^ The largest pore size is related to vascular porosity (VP), which consists of the volume of all tunnels in the bone that contain blood vessels, including all bony canals (primary and secondary) as well as transverse (Volkmann) canals. Lacunar-canalicular porosity (LCP) includes the second-largest porous structure associated with osteocytic lacunae and canalicular channels. The glycocalyx and interstitial fluid of the osteocyte fill the space between the osteocytes and the lacunar-canalicular walls. Finally, the smallest pore size in bone exists in the collagen-apatite porosity (CAP). Most of the water is bound to ionic crystals in the bones at this level.^109^ Oxygenated and nutrient-rich blood passes through the bone capillaries. Blood components then leave with less oxygen, nutrients, and cellular wastes. Various substances, including glucose, amino acids, fatty acids, hormones, neurotransmitters, and inorganic compounds, are exchanged from capillaries into the interstitial fluid in the VP. LCPs are occupied by osteocytes, connecting neighbor cells with elongated cell processes, thereby permitting communication between bone cells. Due to the small pore size and low permeability of LCP (10^−20^ to 10^−25^ m^2^), the lacunar-canalicular system has dramatically higher fluid pressure than VP (similar to blood pressure), leading to a longer relaxation time (~10^−6^ s) compared with VP (~10^−3^ s) after pressure pulse.^51^ In situ measurement of solute transport in the bone lacunar-canalicular system has provided direct evidence for load-induced fluid flow in real-time within the lacunar-canalicular system.^110,111^ The interstitial fluid flow in the lacunar-canalicular system could be enhanced by everyday mechanical loading.^112,113^

**Figure 3.**
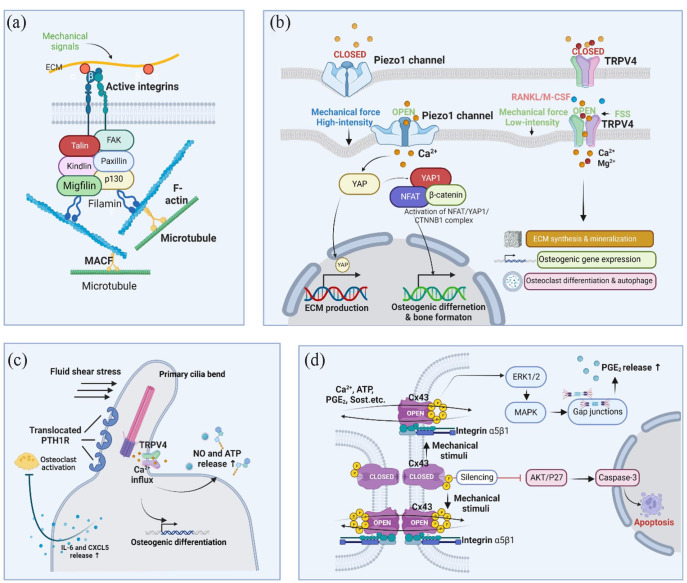
Mechanosensitive structures. (a) Focal adhesions. Focal adhesions connect ECM mechanical signals to the cytoskeleton, affecting cytoskeleton arrangement and crosslinking; (b) Piezo1 and TRPV4. Activation of ion channels by mechanical stimuli elicits specific ion flow, especially calcium influx, to modulate downstream signaling pathways and cell differentiation; (c) Primary cilium. When primary cilia bend under FSS, TRPV4 ion channels open, allowing Ca^2+^ influx and MSCs osteogenic differentiation. PTH1R translocation on primary cilia prevents osteoclast activation by releasing IL-6 and CXCL5; (d) Cx43. When osteocytes experience mechanical stimulation, the Cx43 protein is phosphorylated, and the connexon is opened, allowing the exchange of several effectors, such as calcium, ATP, PGE2, and Sost, between connecting cells through gap junctions. Osteocytes with Cx43-silencing undergo apoptosis via AKT/P27/Caspase-3 pathway. The graph was created with BioRender.com.

Mechanoreceptors sense various external and internal mechanical forces. Detecting external mechanical signals requires mechanoreceptors to be in direct contact with the external environment or to sense changes in intermediate cellular structures (e.g. cell membrane, intracellular plasm movement, etc.) caused by tension, pressure, and FSS. Various cell surface proteins or membrane structures, including focal adhesion, ion channels, connexons, G protein-coupled receptors (GPCRs), and primary cilia, have been identified as potentially mechanosensitive structures (Figure 3). These structures can directly sense single or multiple mechanical signals and change their conformation or activity in response to mechanical stimuli to activate downstream signaling pathways and guide cell behaviors. Below, we discuss these mechanosensitive structures, downstream signaling pathways, and corresponding cellular behaviors in bone. Considering the critical role of osteocytes and FSS in transforming macroscopic mechanical loading to the cellular level, we mainly focus the osteocytes and FSS-related mechanosensation and mechanotransduction.

#### Mechanosensitive structures

##### Extracellular matrix

The fate and function of bone cells are influenced by their niche, which consists of extracellular matrix (ECM) components and surrounding cells. The ECM contains various molecules, such as collagen, fibronectin, elastin, laminin, glycosaminoglycans, and glycoproteins. It provides the cell with a 3D microenvironment, variable stiffness, and signaling molecules. The mechanical properties of the ECM play a significant role in osteocyte behavior. A compact preosteoblast-derived matrix (PDM) can promote the maturation of osteoblasts, whereas loose PDM contributes to the overactivation of osteoclasts.^114^ Changing the stiffness of the matrix can induce osteogenic differentiation of adipogenic human mesenchymal stem cells (hMSCs).^115^ Precise regulation of calcification and elongation is crucial for osteocytes, which are embedded in a bone matrix and extend through the LCP network with cell processes. Osteocytes on a stiffer bone matrix (mineralized) tend to pull more than those on a softer matrix (as-build osteoid), leading to increased tension on stress-bearing elements such as F-actin.^59^ F-actin acts as a mechanosensor, mechanotransduction effector, and primary regulator of YAP (Yes-associated protein) and TAZ (transcriptional coactivator with PDZ-binding motif).^116^

##### Focal adhesion

Focal contacts are direct mechanical linkers between the extracellular matrix (ECM) and the cell, formed by focal adhesion kinase (FAK), integrins, cadherins, and other ECM and cytoskeletal proteins (Figure 3(a)). These contacts facilitate the transfer of signals from the external matrix to the cytoskeleton, promoting cell adhesion, stretching, and migration. Integrins are transmembrane receptors consisting of alpha and beta subunits that form heterodimers. They serve to connect the extracellular matrix (ECM) to the cytoskeleton, enabling the transmission of mechanical stimuli from the extracellular to the intracellular components.

Human primary bone cells express several integrin subunits, including α1, α2, α3, α4, α5, α6, αv, β1, β3, and β5.^117,118^ The α2, αv, β1, and β3 subunits have been proven to participate in sensing mechanical stimuli.^119,120^ Integrin heterodimers possess specific affinities for extracellular matrix (ECM) ligands like collagens, fibronectin, laminin, and other non-collagenous proteins. The aggregation of Integrins promotes the activation and phosphorylation of FAK, which facilitate intermediate proteins like MAPK/ERK/JNK and GTPases to mediate mechanotransduction.^121^ In vitro studies revealed that the blockade of integrin αvβ3 in osteocytic MLO-Y4 cells reduced their sensitivity to the stimulation of laminar oscillatory fluid flow, resulting in impaired COX-2 and PGE2 production.^122^ FSS regulates the activity of the RUNX2 transcription factor by ERK activation, leading to the upregulated integrin β1 expression in hMSCs via the NF-kB pathway.^123^ Integrins are also highly expressed in osteoclasts (αvβ3 and α2β1), but it is unclear whether they are mechanosensitive therein.^124,125^ In vivo studies show that integrin β1 conditional knockout (CKO) mice did not experience bone loss compared to wild-type mice in response to mechanical unloading.^126^ Similarly, mice with CKO of OPN, a ligand for integrins in the ECM, also showed resistance to mechanical unloading-induced bone loss, indicating the significance of the interaction between integrins and their ligands in the bone matrix for mechanosensation and signal transduction.^127^

FAK is a protein that integrates extracellular stimuli with intracellular events and senses mechanical forces generated inside or outside the cell.^128^ Loss of FAK impairs focal contact turnover and disrupts intracellular microtubule polarization via FAK-mediated regulation of Rho-family GTPases.^129^ Rho family GTPases control the assembly and disassembly of the actin cytoskeleton. The RhoA/ROCK pathway involves multiple mechanosensitive signaling pathways, downstream-related ERK activity regulation, and osteogenic differentiation.^130^ Activated RhoA signaling can activate the p38/MAPK and Akt signaling pathways, creating a link between integrins and phosphoinositide 3-kinase (PI3K)/MAPK signaling.^131^ In mandibular stem cells, FAK-mediated mechanotransduction activates new bone formation.^132^ FSS dephosphorylates FAK and inhibits the phosphorylation of histone deacetylase (HDAC) 5 tyrosine 642, which inhibits the expression of sclerostin (*Sost*) in bone cells via an epigenetic mechanism.^133^ FAK catalytic inhibitors can similarly reduce *Sost* expression in vivo and in vitro.^133^ Sost, as a BMP antagonist, can bind to BMP receptors and reduce BMP signaling activity, thereby inhibiting the mineralization functions of osteoblasts.^134^ These findings indicate that FAK is crucial in bone remodeling in response to mechanical loading.

##### Ion channels: PIEZO and TRPV4

Bone is highly responsive to mechanical stimuli, and recent research has highlighted the potential role of PIEZO proteins in skeletal mechanosensation. PIEZO1 and PIEZO2 are mechanosensitive cation channels with similar structures but only 42% sequence identity^135^(Figure 3(d)). In vitro studies have shown that PIEZO1/2 stimulates calcineurin by activating Ca^2+^ influx in osteoblasts, resulting in the coordinated activation of NFATc1, YAP1, and β-catenin in response to mechanical loading.^136^ In vivo studies have demonstrated the crucial role of PIEZO1 in the osteoblast lineage. Reduced protein levels of PIEZO1 and several single nucleotide polymorphisms (SNPs) may be associated with osteoporosis and fractures.^137^ A PIEZO1 CKO (osteoblastic lineage) mouse model confirmed that loss of PIEZO1 impairs bone formation by inhibiting the expression of RUNX2, type I collagen, and OCN.^138^ Moreover, osteoclasts are overactive in Prx1-Cre and Dmp1-Cre guided PIEZO1 CKO (osteoblastic linage) mice, leading to dysregulated interactions between osteoblasts and osteoclasts and subsequent bone loss.^139,140^ Although PIEZO1 and PIEZO2 share similar structures, their mechanosensory functions in bone are not identical. Loss of both PIEZO1 and PIEZO2 results in severe bone defects, whereas loss of PIEZO2 alone has minimal effects on bone, indicating that PIEZO1 is critical for mechanosensation in bone.^136^ However, PIEZO2 has been reported essential for the Merkel-cell mediated mechanotransduction (gentle touch) and proprioception.^141,142^

TRPs are a family of nonselective cation channels that play a crucial role in bone mechanosensation. Among the seven subgroups of this superfamily, TRPV4 is a significant regulator of bone metabolism, determining bone strength and potentially predicting the risk of fractures.^143^ TRPV4 can mediate mechanosensation in osteocytes, chondrocytes, and epithelial cells.^144–146^ Lee et al. reported that FSS in the lacunae activates TRPV4 (not PIEZO1) to increase calcium concentrations in the cellular plasma, accelerating collagen deposition and mineralization.^144,147^ TRPV4 is also involved in mediating oscillatory FSS and laminar shear stress-induced calcium signaling and osteogenic gene expression in bone marrow stromal cells (BMSCs).^144,147,148^ The mechanosensitivity of PIEZO1 and TRPV4 varies with the intensity of mechanical stimuli, with high-intensity mechanical stimuli recognized and input by PIEZO1 and low-intensity mechanical stimuli by TRPV4 (Figure 3(b)).^149^ Notably, TRPV4 is predominantly localized in regions with primary ciliary structures and loses its mechanosensitive in BMSCs with defective primary cilia.^147^ TRPV4 is also expressed in osteoclasts, where it manipulates autophagy and activates NFATc1 signaling to regulate terminal differentiation through Ca^2+^ influx.^150,151^ In the mouse model, TRPV4-knockout leads to marked resistance to hindlimb unloading by inhibiting the increase in bone resorption,^150^ suggesting that such resistance may attribute to TRPV4-deficiency-mediated dysfunction of osteoclasts.

##### Primary cilium

The primary cilium is a microtubule-based, antenna-like sensory organelle found in various bone cells, including osteocytes, osteoblasts, and hMSCs.^152^ Primary cilia protrude into the outer space of the cell and perceive mechanical stimuli.^152^ In osteocytes, primary cilia respond to extracellular fluid pulses generated by physical activities. When primary cilia bend, mechanosensitive ion channels, such as TRPV4, are activated, leading to intracellular Ca^2+^ influx, membrane depolarization, and nerve fiber activation, and the cell then undergoes mechanical stimulation^153^ (Figure 3(c)). The formation of primary cilia was positively correlated with the mechanosensitivity of osteocytes, manifested by the more release of ATP and NO by osteocytes as the length of primary cilia increased.^154^ However, Shi et al. reported that a simulated microgravity (SMG) environment abolished primary cilia formation and shortened the residual cilia, inhibiting the formation and mineralization of rat calvaria.^155^ Periosteal osteochondroprogenitors can perceive FSS via primary cilia and differentiate into osteoblasts. This response can be invalidated almost entirely in the absence of primary cilia.^156^ Similarly, the normal osteogenic response to FSS is also reduced in MC3T3-E1 and MLO-Y4 cells after abrogating primary cilia.^157,158^ Osteocytes with PTH1R translocation to primary cilia can prevent osteoclast formation under FSS by manipulating CXCL5 and IL-6 secretion.^159^ Therefore, restoring or enhancing the function of primary cilia may be a potential strategy to combat bone loss associated with mechanical disuse, such as microgravity. Nevertheless, current studies do not support the existence of primary cilia in osteoclasts.^160^

##### Primary cilium

The primary cilium is a microtubule-based, antenna-like sensory organelle found in various bone cells, including osteocytes, osteoblasts, and hMSCs.^152^ Primary cilia protrude into the outer space of the cell and perceive mechanical stimuli.^152^ In osteocytes, primary cilia respond to extracellular fluid pulses generated by physical activities. When primary cilia bend, mechanosensitive ion channels, such as TRPV4, are activated, leading to intracellular Ca^2+^ influx, membrane depolarization, and nerve fiber activation, and the cell then undergoes mechanical stimulation^153^ (Figure 3(c)). The formation of primary cilia was positively correlated with the mechanosensitivity of osteocytes, manifested by the more release of ATP and NO by osteocytes as the length of primary cilia increased.^154^ However, Shi et al. reported that a simulated microgravity (SMG) environment abolished primary cilia formation and shortened the residual cilia, inhibiting the formation and mineralization of rat calvaria.^155^ Periosteal osteochondroprogenitors can perceive FSS via primary cilia and differentiate into osteoblasts. This response can be invalidated almost entirely in the absence of primary cilia.^156^ Similarly, the normal osteogenic response to FSS is also reduced in MC3T3-E1 and MLO-Y4 cells after abrogating primary cilia.^157,158^ Osteocytes with PTH1R translocation to primary cilia can prevent osteoclast formation under FSS by manipulating CXCL5 and IL-6 secretion.^159^ Therefore, restoring or enhancing the function of primary cilia may be a potential strategy to combat bone loss associated with mechanical disuse, such as microgravity. Nevertheless, current studies do not support the existence of primary cilia in osteoclasts.^160^

##### Connexon 43 (Cx43)

Gap junctions act as intercellular channels, facilitating the passive diffusion of small molecules (<1 kDa) and electrical currents between neighboring cells in response to extracellular stimuli (Figure 3(d)). They consist of two docked, hexagonal connexons, each comprising six connexin molecules.^161^ Connexin 43 (Cx43) is a prevalent isoform expressed in humans and rodents’ osteoblasts, osteocytes, and osteoclasts.^162^ Cheng et al. found that both pulsating and steady fluid shear stress (FSS) can induce the redistribution of intracellular Cx43 from the perinuclear region to the cytoplasm and processes of osteocytes.^163^ Osteocytes exposed to FSS at 1.6 Pa (16 dyn/cm^2^) increased Cx43 expression on the cell membrane, leading to the formation of hemichannels, thereby facilitating the release of PGE_2_ and the construction of intercellular gap junctions.^163–166^ Interestingly, mechanical stretching of osteoblasts can promote the phosphorylation level of Cx43 without affecting its mRNA expression.^167^ Furthermore, the oscillating fluid flow facilitates the development of new gap junctions (GJs) between mouse osteocytes by an ERK1/2-MAPK-dependent mechanism, while the dye transfer between existing GJs remains unchanged.^168^ However, Cx43 cannot sense mechanical stress independently but requires interaction with conformationally activated integrin α5β1 (C-terminal) to open the Cx43 hemichannel.^169^ These stress-sensing structures work together to enhance cellular mechanosensitivity. The findings suggest that Cx43 and integrin α5β1 are tightly coordinated in sensing mechanical stimuli and improving cellular mechanosensitivity.

Several studies have emphasized the importance of Cx43 in normal bone formation. Cx43-silenced MLO-Y4 cells underwent apoptosis through the AKT/P27/Caspase-3 pathway.^170^ However, different Cx43 CKO mouse models have yielded different conclusions. Specifically, Col1-Cre or Dmp1-Cre-guided Cx43 CKO resulted in bone loss, impaired osteoblast function, and reduced mechanical loading-mediated bone anabolism.^171–173^ In contrast, an Ocn-Cre-guided osteocyte/osteoblast Cx43 CKO mouse model showed increased osteolytic function by manipulating the RANKL/OPG ratio and enhancing anabolic responses mediated by mechanical loading.^174^ Another study found that CKO of Cx43 in osteocytes and osteoblasts prevented mechanical unloading-mediated loss of trabecular bone but failed to maintain the mechanical properties of cortical bone without suppressing cortical bone formation.^175^ Interestingly, Cx43 CKO in osteocytes (guided by Dmp1-Cre) enhanced the mechanoresponsiveness in mice.^172^ Compared with WT mice, Cx43 CKO mice exhibited a higher rate of periosteal bone formation, manifested by elevated mineralized surface and enhanced mineral deposition rate, which may be due to the loss of Cx43 in osteocytes promoting stretch-induced expression of β-catenin and its target genes.^172^ The complicated results from different systems may attribute to the unspecific CKO cell coverage guided by different Cre molecules and the dual functions of Cx43 in gap junctions and hemichannels.

##### GPCRs

G protein-coupled receptors (GPCRs) have been proposed as mechanosensitive structures. However, only specific GPCRs can sense mechanical forces, such as Angiotensin II receptor 1 (AGTR1), bradykinin receptor B2 (BDKRB2), and GPR68.^176,177^ Mechanosensitivity is determined by the presence of the C-terminal helix 8 (H8) domain, which is absent in mechano-insensitive GPCRs but can be linked to confer mechanosensitivity.^178^ The activation pathway of GPCRs involves agonist binding and subsequent conformational change, activating guanine nucleotide exchange (GEF) activity toward one of the potentially interacting heterotrimeric Gαβγ protein elements. Then, GDP on the α subunit is replaced by GTP, leading to the activation and dissociation of Gα from the βγ subunit. The activated α/β/γ subunit activates different downstream effectors, such as phospholipase C, adenylyl cyclase, GIRK channels, and PI3K.^179^ Mechanosensitive GPCRs can induce downstream signaling events upon activation by mechanical stress, including increases in intracellular calcium concentrations via PLC-IP3 and DG pathways.^180^ GPCRs often function as a multi-sensitive surface structure. GPR68, for example, responds to extracellular acidification during membrane stretching. Its activity level reflects the degree of membrane stretching and acidification.^181^ Further research is needed to comprehend their involvement in physiological and pathological states within bone remodeling.

#### The mechanotransduction pathways

**Figure 4.**
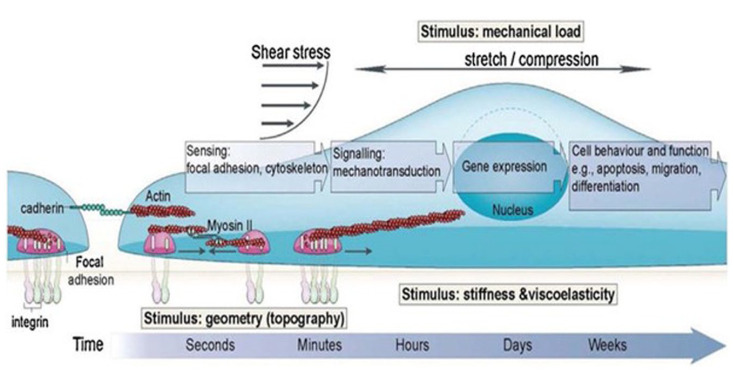
Showing biological response to different mechanical stimuli to regulate cell function and behavior. Figure reprinted from Iskratsch et al.^185^ with permission under license number 501718235. Copyright © 2014, Nature Publishing Group. All Rights Reserved.

Mechanotransduction converts physical load to biochemical signals,^182^ which change the morphology and function of cells, gene expression, and ECM synthesis.^183^ This process involves four steps: (i) mechanocoupling, (ii) biomechanical coupling, (iii) transmission of signals from the sensor cells to the effector cells, and (iv) responses of the effector cells.^184^ As illustrated in Figure 4, this process involves receptors (e.g. cadherins and integrins), mechanosensors (e.g. stretchable proteins such as p130CAS and talin), and nuclear cues factors, which alter protein and gene expression profiles. Other factors, such as gender and age, can also regulate the mechanotransduction process.^185^ For example, the impact of age has been investigated previously; research on rats of diverse ages showed that inducing bone formation in older rats was over 16-fold less than in younger ones by applying a load of 64 N. Thus, age can be considered an inhibitory factor of bone formation.^184^ Gender also acts as a contributor since men have less mechano-responsiveness than women.^186^

When a load is applied to the bone, osteocytes detect the fluid flow and then generate and transmit signaling molecules that modulate the osteogenic/osteoclastic functions of osteoclasts and osteoblasts, respectively, thereby affecting bone remodeling consequently.^60^ Mechanotransduction has been investigated from two perspectives: the micro level and the macro level. The macro perspective deals with the system that mechanical stimuli can form by compression or stretching on cells between neighboring cells or cell membrane interfaces. Mechanotransduction from a macroscopic perspective involves mechanical loads imparting varying degrees of deformation to the bone matrix through compression and stretching or imparting fluid flow to the lacunae-tubular network, transmitting mechanical stimuli to mechanosensitive cells represented by osteocytes. On the other hand, the micro perspective has sought to focus on characterizing specialized molecular signaling pathways in specialized tissues. Both these perspectives are involved in particular theories.^187^ The one theory investigating the impact of mechanical stresses on the living cells’ function and molecular structure is tensegrity. Tissue and living cells use a form of architecture called tensegrity.^188^ The factors involved in this theory are structure (3D structure) and the prestress level.^189^ This type of architecture obtains its mechanical stability via the transmission of continuous tension by the geodesic path and through an internal prestress’ presence. Regarding living cells, internal compression elements create this prestress that resists the inward pull of surrounding tensile actomyosin filament networks.^188^ Therefore, it can protect the cells against damage by disturbing the forces; furthermore, a mechanical stimulus, even on a small scale, can affect many cells and various cellular functions.^190,191^ In this theory, the above-mentioned focal adhesions, integrins, ion channels, connexons, primary cilia, and GPCRs are considered to mediate the mechanosensation of bone cells. At the same time, multiple pathways or mechanisms are involved in intracellular mechanotransduction and corresponding functional responses, including cytoskeleton, RhoA/ROCK, YAP/TAZ, etc.

##### Cytoskeleton

Cytoskeleton is a fibrous network formed by the nuclear skeleton, cytoplasmic skeleton, cell membrane skeleton, cross-linking factors, and extracellular matrix. It provides the framework of basic cell morphology and connects all mechanosensitive components. Among the main cytoskeletal elements, F-actin can sense and transmit mechanical stimuli in osteocytes.^59^ Myosin II acts like a cross-linker, strengthening or softening the actin network by directing filament sliding, disassembly, and rearrangement.^192,193^ This ability to stiffen or soften provides cells with an intrinsic mechanism for maintaining global morphology in response to mechanical stimuli in different magnitudes. Myosin II activity is determined by the phosphorylation of its light and heavy chains mediated by multiple kinases, which are activated by Ca^2+^ (MLCK), RhoA(citron kinase), Cdc-42 (myotonic dystrophy kinase-related Cdc-42-binding kinase, MRCK).^194^ As a cytoskeletal linker between F-actin and microtubules (Figure 3(a)), microtubule-actin cross-linking factor 1 (MACF1) is known as a mechanosensitive structure due to its reduced expression in response to mechanical unloading both in vitro and in vivo.^195^ MACF1 mediates the phosphorylation of EB1 at Y247. p-EB1 moves along microtubular bundles, contributing to the polarization, motility, and focal adhesion turnover of pre-osteoblasts.^196^ Hu et al. reported that MACF1 significantly enhances the mineralization of MC3T3-E1 cells by promoting the β-catenin/TCF1-RUNX2 signaling pathway.^147,197^ However, loss of MACF1 results in dysfunction of microtubule organization.^143,144^

The cytoskeleton determines bone cell morphology and mechanosensitivity. Cultured osteocytes in round shape appear more responsive to mechanical stimuli than adherent flat osteocytes (MLO-Y4).^198^ The less stiff cytoskeleton of round cells may facilitate the response of cells to tiny strains mediated by mechanical loading.^198^ However, because dendritic osteocytes in the lacunar-canalicular system can amplify and perceive the micro-deformation of bone tissue, the significance of this low-stiffness cytoskeleton in the round cells to bone health needs further study. Microgravity (as well as SMG) leads to cytoskeleton depolymerization and misarrangement of microfilaments and microtubules.^199^ In osteoblasts, cytochalasin B-induced SMG impedes BMP2-induced Smad1/5/8 activation and RUNX2 expression by hindering the F-actin polymerization.^200^ Our recent study also showed that nanotopography-mediated M1 polarization of human primary macrophages on Titanium implants was impaired under an SMG environment (induced by cytochalasin D), suggesting that F-actin plays an essential role in the mechanosensation/mechanotransduction of macrophages.^201^ The crosstalk between BMSCs and M1/M2 polarized macrophages can further manipulate the balance of osteogenesis and osteoclastogenesis in the local milieu, ultimately determining the outcome of bone remodeling.^202^

##### RhoA/ROCK

Small GTPases undergo conformational changes between their active GTP-bound and inactive GDP-bound states to transduce information through signaling pathways. Such process is accelerated by GEFs and GTPase activating proteins (GAPs), which assist GDP dissociation and GTP hydrolysis, respectively.^203^ In addition, guanine dissociation inhibitors (GDIs) can bind to small GTPases and redistribute them to the membrane or cytoplasm.^203^ The most well-studied GTPases include RhoA, Rac1, and Cdc42.^204^ As a member of the Rho family of 20 small GTPases encoded in mammalian genomes,^205^ the RhoA signaling pathway is essential for mechanotransduction as it regulates the response of the actin cytoskeleton to mechanical forces.^206^ The activation and inactivation of RhoA are controlled by upstream signals from various receptors, including GPCRs, integrins, and growth factor receptors (TGF-βR). Mechanical stimuli such as FSS can activate small RhoA via a GEF-dependent mechanism. GEF binds to the inactive RhoA-GDP to form a RhoA-GEF dimer, which promotes the dissociation of GDP from Rho and facilitates the binding of GTP, leading to RhoA activation. Activated RhoA then interacts with its essential effectors (Rho-associated protein kinase family, ROCK; particularly ROCK1 and ROCK2) and phosphorylates myosin phosphatase, resulting in the contraction of the actin cytoskeleton by activating myosin light chain.^207,208^

RhoA/ROCK2 regulates the osteogenic differentiation of C3H10T1/2 cells and MSCs and has additive effects on *RUNX2* expression under oscillatory fluid flow.^207,209^ Myocardin-related transcription factor (MRTF) and YAP/TAZ have been identified as transcription factors activated by mechanical stimulation.^210^ When external forces or endogenous cell stress act on the cell, the mechanosensor is stimulated by the cytoskeleton and cell membrane tension, leading to the activation of related pathways and changes in gene expression through Rho/ROCK mediated activation of actin-MRTF-serum response factor (SRF) signaling pathway.^211,212^ Stretching can activate the RhoA/ROCK signaling pathway and YAP/TAZ, resulting in the polymerization of F-actin, promoting osteogenic differentiation of MSCs while inhibiting adipogenic differentiation.^213^ A similar RhoA-YAP/TAZ pathway also participates in sensing and transducing the ECM stiffness signals, thereby manipulating the mechanosensitivity of osteoblasts through cytoskeleton reorganization.^214^ Moreover, the activation of P2Y_2_ receptors mediated by FSS regulates the mechanosensitivity of MC3T3-E1 cells via RhoA/ROCK signaling pathway.^215^

##### YAP/TAZ

The Hippo pathway regulates crucial cellular processes through YAP and TAZ activity by integrating various signals.^216^ YAP and TAZ are transcriptional coregulators lacking a DNA-binding domain, necessitating their interaction with DNA-binding proteins to regulate transcriptional activity. The Hippo pathway can limit tissue growth and cell proliferation by phosphorylating YAP/TAZ. In mammals, SAV1 and MST1/2 form heterodimers that phosphorylate SAV1, MOB1, and LATS1/2 kinases, leading to direct phosphorylation of YAP and TAZ at multiple sites via LATS1/2.^217^ Then, the phosphorylated YAP/TAZ is trapped in the cytoplasm and undergoes degradation through the ubiquitin-proteasome system.^218,219^ Conversely, when the Hippo pathway is off, YAP/TAZ are kept dephosphorylated and translocated into the nucleus, interacting with co-transcriptional factors to initiate transcriptional programs associated with cell proliferation, survival, and migration.^220,221^

Various upstream inputs regulate the nuclear localization of YAP/TAZ in response to mechanical stresses. Low stiffness increases intracellular phosphatidylinositol 4,5-bisphosphate and phosphatidic acid levels through phospholipase Cγ1 (PLCγ1), which activates RAP2, a Ras-related GTPase to relay ECM rigidity signals and control the mechanosensitive cellular activities.^222^ RAP2 triggers the LATS1/2 activation, leading to the phosphorylation and degradation of YAP/TAZ.^222^ In cells experiencing low mechanical signaling, the ARID1A/SWI/SNF-YAP/TAZ complex inhibitory interaction also predominates. Conversely, nuclear F-actin binds to ARID1A/SWI/SNF at high mechanical stress, preventing the formation of the ARID1A/SWI/SNF-YAP/TAZ complex and promoting YAP/TAZ association with TEAD (their DNA binding platform).^223,224^ It is reported that ECM with high stiffness increases the abundance of vinculin, which promotes the nuclear accumulation of YAP/TAZ independent of LATS1 and following osteogenic differentiation of MSCs.^225^ Vinculin deletion with shRNA abrogates rigid ECM-mediated osteogenic differentiation of MSCs while promoting adipogenic differentiation.^225^ Therefore, promoting YAP/TAZ nuclear accumulation by inactivating Hippo signaling and enhancing YAP/TAZ binding to TEAD by genetically deactivating ARID1A/SWI/SNF or raising cellular mechanics may be effective strategies to strengthen the responsiveness of YAP/TAZ to mechanical stimuli.

YAP/TAZ in osteocytes is crucial for maintaining bone mass and regulating matrix collagen content and organization, affecting bone mechanical properties.^226^ In a recent study, Zarka et al. investigated the significance of YAP/TAZ in osteocyte mechanotransduction. They found that YAP/TAZ translocated to the nucleus and activated their target genes in 3D cultured osteocytes under mechanical compression.^227^ Silencing of YAP/TAZ with shRNA partially blocked the mechanical-loading-induced M-CSF and Cxcl3 genes expression, indicating that YAP/TAZ function as a mediator of mechanically-induced chemokine expression in osteocytes.^227^ Furthermore, transcriptomic analysis of YAP/TAZ-depleted osteocytes under compressive strain revealed several key factors in initiating dendrites formation associated with YAP/TAZ.^227^ These findings suggest that YAP/TAZ plays a central role in forming the perilacunar/canalicular network and osteocyte-mediated mechanotransduction/bone remodeling.

YAP and TAZ play intricate roles in osteogenesis. TAZ is generally considered a transcriptional coactivator that interacts with Runx2 and serves as a key regulator of osteoblastogenesis.^228^ siRNA silencing of TAZ abolishes osteogenic differentiation induced by FGF-2 and IGF-1 in cultured rat bone marrow. In contrast to TAZ, YAP inhibits Runx2 activity in ROS 17/2.8 osteoblast-like cells and regulates osteoblastogenesis through Wnt/β-catenin signaling in vitro and in vivo.^229,230^ SMG significantly weakens the osteogenic differentiation of rat MSCs via the downregulation of TAZ activity. However, by activating ROCK signaling, TAZ activated by lipophosphatidic acid can counteract the inhibitory effects of SMG on osteogenic differentiation in MSCs.^231^ Recent studies have utilized mouse models to investigate the roles of YAP/TAZ in bone formation and have revealed their diverse functions depending on the stage of osteoblastogenesis. Induction of YAP/TAZ double deletion in Prx1^Cre^ MSCs was found to promote osteoblastogenesis and bone formation in 12-week-old mouse vertebral cortical bone.^232^ Conversely, conditional deletion of YAP in fully differentiated osteoblasts in YAP^fl/fl^-Ocn^Cre^ mice resulted in bone loss due to decreased osteoblast proliferation and differentiation.^232^ Furthermore, YAP^fl/fl^/TAZ^fl/fl^-Osx^Cre^ mice showed increased osteogenic differentiation with upregulated Osx, osteocalcin, and collagen I levels. Such double deletion-induced enhancement of osteogenesis was associated with activation of the Wnt/β-catenin signaling and increased *Runx2* expression.^232^ However, YAP or TAZ single knockout in Osx^+^ cells or YAP/TAZ double knockout at the mature osteoblast/osteocyte stage (YAP^fl/fl^/TAZ^fl/fl^-Dmp1^Cre^) led to decreased bone formation and increased osteoclast activity.^232,233^ In summary, YAP/TAZ can promote osteogenic activity in fully differentiated osteoblasts/osteocytes while inhibiting the commitment of stem cells into the osteoblastic lineage.

##### Wnt/β-catenin

The Wnt signaling pathway has diverse functions in bone remodeling and homeostasis.^234^ Canonical Wnt signaling is triggered by the binding of Wnt ligands to Frizzled and Lrp5/6 receptors on the cell membrane. This signaling promotes β-catenin accumulation by inhibiting GSK-3β-induced β-catenin phosphorylation, and translocated β-catenin then induces transcription of LEF/TCF-responsive genes.^235^ β-catenin is a critical mediator of mechanotransduction, and its activity is modulated by mechanical loading and unloading via activation of the nitric oxide, FAK, and Akt signaling pathways.^236^ Strength and power training can increase Wnt-related gene expression in human subjects, while mechanical strain induces MSCs to switch from adipogenic to osteogenic differentiation by preserving β-catenin in the nucleus.^237,238^

In osteocytes, Wnt/β-catenin signaling plays a vital role in mechanotransduction. Mice with β-catenin deletion in osteocytes exhibit severe osteopenia and fragile bones.^239^ Wnt signaling-activated transgenic mice (LRP5 G171V) show upregulated Wnt/β-catenin target gene expression and increased bone formation under physiological and mechanical loading conditions.^240^ Conversely, the absence of Wnt inhibitors (FRZB and Sost) enhances the anabolic activity of bone in response to mechanical loading.^241,242^ Furthermore, mechanical loading promotes Postn expression and inhibits Sost expression through the Postn-integrin αVβ3 interaction, while unloading produces the opposite effect.^243^ However, high-intensity mechanical loading can inhibit the PI3K/Akt pathway, leading to β-catenin phosphorylation and impaired osteoblast differentiation.^244^ Mechanical loadings can also activate non-canonical Wnt signaling. Oscillating fluid flow induces the expression of Wnt5a and its non-canonical tyrosine kinase receptor Ror2, which are required for mechanically mediated RhoA signaling activation and osteogenesis.^245^ Overexpression of Ror2 enhances osteogenesis, indicating that non-canonical Wnt signaling plays a crucial role in mechanotransduction.^246^ These findings support the involvement of canonical and non-canonical Wnt signaling in bone mechanotransduction and provide insights into the mechanisms underlying the effects of mechanical loading on bone remodeling.

##### Potential pathways and mediators

Various signaling pathways and factors have been discovered to mediate the transduction of mechanical signals in bone cells, in addition to the molecules and pathways previously mentioned. One of these is the Ras/ERK-mediated mitogen-activated protein kinase (MAPK) signaling, which can be activated by mechanical forces, promoting hypoxia-inducible factor 1-alpha (HIF-1α) expression in osteoblasts.^247^ Osteoblast-targeted delivery of miR-33-5p, a noncoding RNA, has been found to enhance osteogenesis and partially counteract the reduction of osteogenic genes and mineral apposition rate in the hindlimb unloading mouse model.^248^ Furthermore, during the commitment of hMSCs to the osteogenic lineage, cell shape has been observed to modulate the ability of BMP2 to activate RhoA, ROCK, and cytoskeletal tension. RhoA/ROCK activity and associated cytoskeletal tension can regulate hMSC commitment to the BMP-induced osteogenic phenotype.^249^ As further studies are conducted, more transcription factors involved in metabolic and hypoxic modulation in response to mechanical loading are expected to be identified. HIF-1α CKO in osteoblasts has been reported to result in the formation of thinner cortical bone, highlighting the importance of such factors in the process of bone formation.^247^ The epigenetic mechanism also involves the mechanical loading mediated bone formation. In MSCs with osteogenic differentiation induced by cyclic stretching and compression loading, histone deacetylase (HDAC) activity decreases, accompanied by increased histone acetylation and remodeled chromatin. Deleting nuclear matrix protein lamin A and C abrogates mechanical loading-induced alteration in histone acetylation.^250^

## Effect of mechanical loading on bone healing and regeneration

Bone healing and regeneration involve a variety of bone defects, including fractures, traumatic bone defects, and medical-related bone injuries (implantation of endosseous medical devices), which have different mechanical properties. In this section, the in vivo evidence and the in vitro mechanism research data on the influence of mechanical loading on the healing of fracture and bone trauma were summarized and analyzed to obtain potential clinical intervention strategies.

### Mechanoresponses of bone healing/regeneration in vivo

A vast diversity of mechanical factors has been recognized to affect fracture healing. The predominant factors in this process include rigid fixation, fracture geometry, fracture type, direction, and magnitude. All these factors determine local stress distribution at the fracture site and provide mechano-biological signals to regulate fracture healing and elicit cellular reactions.^10^ Not only the amount of interfragmentary movement but also its direction influences the healing process. Moderate axial interfragmentary movement enhances fracture repair by promoting periosteal callus formation and accelerating healing.^251^ Conversely, tensile or shear movements of similar magnitude do not appear to promote fracture healing. While induced cyclic tensile strains can stimulate periosteal callus formation but fail to promote bone healing.^252^ Shear movements at the fracture site have been shown to impede healing, manifested by decreased periosteal callus formation, delayed bone formation in the fracture gap, and inferior mechanical stability compared to the axial movement in a sheep model after loading (immediate post-surgery to 8 weeks).^253^ However, in a clinical case, the shear movement induced by 15 kg loading 2 weeks (full body weight applied after 8 weeks) after closed, low-energy diaphyseal tibial fractures is shown to be compatible with successful healing.^254^ In vivo investigation in rabbit model also demonstrated that shear movement resulted in superior healing outcomes 4 weeks after fracture but inferior outcomes 2 weeks after fracture compared to axial interfragmentary movement. Such shear movement-induced improvement in fracture healing occurs through enhanced endochondral ossification.^255^ Therefore, the shear movement appears more sensitive to timing, magnitude, and gap geometry than axial movement.

Liu et al. investigated the impact of the timing phase of force application on bone defect healing in a mouse model. This study showed that applying daily loading of 5 N peak load, 2 Hz, 4 consecutive days, 60 cycles within inflammation and hematoma consolidation disrupted the traumatic site and activated cartilage formation surrounding, which impedes stabilization of the trauma site. On the contrary, loading throughout the matrix deposition phase improved cartilage and bone formation; Loading within the matrix deposition phase enhanced both bone and cartilage formation; Loading within the remodeling phase increased woven bone formation.^256^ Another rat in vivo study reported the effect of delayed and immediate cyclic axial load (0.05 Hz, 30 g loading with 2.2% graft elongation) on the tendon graft-bone interface healing. The results demonstrated that delayed loading improved biological and mechanical parameters of tendon-to-bone healing compared to immediate loading.^257^ Gardner et al. reported similar results from a mouse model that both timing and loading magnitude affected fracture healing. Compared to the immediate loading model, the low magnitude (0.5 N, 1 Hz for 100 cycles/day, 5 days/week for 2 weeks) axial cyclic compression with a short delay (4 days delay) led to significantly improved fracture healing, evidenced by increased callus strength which vanishes with the increase in loading amplitudes (2 N). Therefore, mechanical loading in inappropriate timing and overloading can potentially impair fracture healing.^258^ Wehrle et al. reported that the bone remodeling (from week 4 to 7) behaviors are more responsive to cyclic mechanical loading. Cyclic strain (8–16 N, 10 Hz, 3000 cycles; 3 times/week for 4 weeks) applied on the mouse fracture model led to significantly higher callus formation and mineralization, which may associate with Wnt signaling activation and reduced distribution of sclerostin and RANKL in fracture callus.^259^ Such time- and magnitude-dependent acceleration of fracture healing may attribute to the enhanced exchange of cells and bioactive factors mediated by loading-mediated callus deformation and altered interstitial fluid flow. Ghimire et al. established a finite elemental model to analyze the impact of dynamic loadings (150 N, 1 Hz for 5 h) on fracture healing (human tibia bone) under various locking compression plate configurations. Dynamic loading increased the transport of bone cells (280% for chondrocytes and 180% for osteoblasts) and growth factors (220% for chondrogenic growth factors and 120% for osteogenic growth factors) in the callus compared to the free diffusion. Similarly, a moderate transport improvement was observed for the MSCs and fibroblasts, around 22% and 17%, respectively.^260^ Another study on the sheep metatarsus fracture model showed that mechanical loading with low amplitude and high frequency (0.02 mm of compression displacement with frequencies between 50 and 100 Hz) significantly improved the osteogenic activity of the callus. Regarding the four mechanical variables (deviatoric strain, octahedral strain, pore pressure, and fluid flow velocity) tested within the callus, only interstitial fluid flow velocity underwent significant increases in amplitude and peak value when the frequency of the external stimulus was altered.^261^ The regulation of bone formation by mechanical loading is also influenced by overall health status. An in-depth study conducted by Maycas et al. applied the combination of parathyroid hormone-related protein (PTHrP)-derived peptides and mechanical loading to treat skeletal deterioration in a diabetic mouse model. In diabetic mice, mechanical loading induced less bone formation than in healthy mice. The combination of mechanical stimuli and PTHrP peptide can overcome bone loss, fragility, and reduced mechanoresponsiveness caused by diabetes.^262^ Li et al. also reported the impact of spinal compression loading (4 N, 10 Hz, 5 min/day for 2 weeks) on bone formation in ovariectomized (OVX) mice. The results supported the hypothesis that Wnt3a-mediated signaling was involved in the effects of spinal loading on enhancing bone formation/angiogenesis and repressing bone resorption in OVX mice. Wnt3a may work as a potential mechanosensitive therapeutic target for postmenopausal osteoporosis.^263^ Applying bend loadings (31, 43, 53, and 65 N, single boat for 36 or 360 cycles) also facilitated bone formation at the endosteal surface. The lamellar bone formation rate (BFR) was enhanced in all categories (Maximum bone formation obtained after loading of 65 N), suggesting that bone lining cells could be stimulated by bend loading and contribute to the anabolic responses on the bone surface.^264^

The type of mechanical loadings is supposed to be crucial for the response of bone. For instance, bone cannot adapt to loading unless applied cyclically (as physiological movement or physical exercises). Hert et al. found that static bending on the tibiae of rabbits for 30 days impaired bone formation. In contrast, rabbits subjected to dynamic loading of the equivalent magnitude were shown to have enhanced bone formation on both endosteal and periosteal surfaces.^265^ Similarly, rats with static loading at 8.5 and 17 N (10 min/day, day 1–5 and 8–12) showed the same bone formation on the periosteal bone surface. Static loading could not generate an anabolic bone response but even suppress the appositional growth of the skeleton with the increase of loading magnitude. However, applying a dynamic force at 17 N (haversine waveform, 2 Hz, 1200 cycles/day) for a similar period significantly enhanced bone formation.^266^ Bone cells can rapidly desensitize under static loading and lose mechanosensitivity before mechanosensation and mechanotransduction are complete.^267^ Therefore, cyclic and intermittent loading may be more beneficial for maintaining bone mechanosensitivity than continuous loading because more rest phases are presented.^268^ Furthermore, if loading cycles are divided into discrete bouts with hour intervals, the mechanical loading protocol may be more osteogenic than the cycles applied within one uninterrupted bout. Robling et al. evaluated the effect of discrete mechanical loading bouts on the biomechanical and structural properties of the rat ulna. The right ulnas of 26 adult female rats were exposed to a haversine waveform at 17 N peak value, 360 cycles/day, 3 days/week for 16 weeks. In half of the experimental subjects, all 360 daily cycles were applied in a single bout (uninterrupted, 360 ×1). The other subjects were applied 90 cycles four times per day (90 × 4), with an interval of 3 h between bouts. The loaded ulnas showed 5.4% (360 ×1) and 8.6% (90 ×4) greater areal bone mineral density than the control. Bone mineral content was enhanced by 6.9% and 11.7% in the 360 × 1 and 90 ×4 loaded ulnas, respectively.^269^

In addition to fracture and bone defect healing, osteogenesis in normal bone and the osseointegration of implants have also been shown to be highly dependent on mechanical loading. A comparative rat study evaluated the effect of ultrasound and mechanical compression on normal bone in three different groups: (i) transcutaneous low-intensity pulsed ultrasound (1 MHz sine waves with an intensity of 30 mW/cm^2^) applied on the left ulnae; (ii) ultrasound and compression loading (0.003 at 0.12/s, with a 0.46 s rest period at peak strain and a 10 s rest period between each cycle, 3 times/week for 2 weeks) applied on the left ulna simultaneously; (iii) compression loading applied on the left ulna. *The bone formation was evaluated by measuring the periosteal bone surface by the d*ouble label (dLS/BS, %). *All groups showed a considerably* increased mineral apposition rate (MAR) and enhanced dLS/BS % from less than 10% in the control samples to more than 80% in the treated samples.^270^ The study conducted by Chavarri-Prado et al. provides evidence of the impact of mechanical loads and exercise on osseointegration. Four dental implants were placed in both tibiae of 10 New Zealand rabbits, which were divided into two groups. The test group underwent 20 min of daily treadmill running during the osseointegration period (with a 2-week progressing adaptation phase), while the other group served as control. The test group had more significant vertical bone growth (1.26 ± 0.48vs 0.32 ± 0.47 mm, *p* < 0.001), higher ISQ values (11.25 ± 6.10vs 5.80 ± 5.97 *p* = 0.006), higher BIC (25.14 ± 5.24%vs 18.87 ± 4.45%), and higher bone neoformation (280.50 ± 125.40vs 228.00 ± 141.40 mm^2^, *p* = 0.121).^271^ Zhang et al. evaluated different factors for bone-implant contact (BIC) and a peri-implant bone fraction (BF). In the rat tibia compression model with titanium implant placement, the high and low frequencies (HF, LF) with high and low magnitudes (HM, LM) were applied as follows: HF/LM (40 Hz, 0.5 N); HF/HM (40 Hz, 1 N), LF/LM (2 Hz, 10 N), and LF/HM (2 Hz, 20 N). Both HF/LM and LF/HM effectively improved the BF and BIC at the cortical level. However, BIC at the medullar level was positively influenced only in the case of HF-LM loading.^272^ Such HF/LM-mediated improvement could attribute to changes in the interstitial fluid flow velocity, which promotes endochondral ossification, cell proliferation, migration, and ECM synthesis.^273^ The relationship between the peri-implant bone, osseointegration, and mechanical loading can be found in the animal model and clinical research.^274^ Nevertheless, due to the limit of the device access and ethical issues, experimental mechanical loadings with specific types, magnitudes, and frequencies are hard to be conducted on the clinical implant. On the contrary, there is no difference in the success rate between immediate loading (immediate occlusal vs non-occlusal loading) and conventional loading (3–6 months delay).^274,275^ Occlusal loading does not lead to improved implant osteointegration. On the other hand, immediate loading seems harmful (2.7 times more risk compared with delayed loading) to the survival of implants 1 year after surgery.^276^ According to the studies of mechanical loading with a delay (matrix deposition or bone remodeling phases), more detailed comparative clinical studies are needed to clarify if delayed loading can benefit bone healing around the implant. In strategies to improve fracture healing or implant osseointegration using mechanical stimulation, the priority is maintaining primary stability (bone-implant interface or between fracture fragments). On this basis, high-frequency and low-level loading with resting intervals can be employed to stimulate the osteogenic response of the callus and avoid adverse movement of the trauma site. Since the existing studies are based on different animals and mechanical models, it is difficult to unify the parameters and models of mechanical loading, which is detrimental to forming a theoretical consensus on the biological response of bone to mechanical loading. Finite element analysis and mathematical modeling based on big data help to obtain more uniform mechanical parameters. In addition, additional systemic factors should be considered, especially in elderly patients with reduced mental performance and coordination. These patients require early mobilization but are often unable to avoid uncontrolled full weight bearing and thus may experience adverse interfragmentary motion. The porotic bone of the elderly will also increase the shear movement (Table 1).

**Table 1.** The summary of in vivo studies analyzed in this review on the impacts of mechanical loading on fracture healing/regeneration.

Mechanical loading model and parameters	Main findings	Immediate /delay loading post-trauma
*Fracture healing*		
Clinical trial with axial sliding model (82 human subjects). 1st stage: axial displacement (1 mm, 0.5 hz, 20 min/day); 2nd stage: axial movement allowed over the level of 12 kg.	Both clinical healing and mechanical healing were enhanced in the group subjected to axial micromovement, compared to the control group in a fixed mode	Delay (0–7 days)^251^
Clinical trial with diaphyseal tibial shaft fractures (3 volunteers, 2 oblique fractures and 1 transverse fracture). 1st stage: 15 kg loading between week 2 and 8; 2nd stage: full body weight loading.	No difference between transverse fractures and oblique fractures. The shear movement induced by 15 kg loading is shown to be compatible with successful healing	Delay (2 weeks)^254^
Sheep fix-sliding model with diaphyseal osteotomy (24 subjects). Nonuniform cyclic tensile strains (0.2 and 0.8 mm displacement, 1,5 and 10 Hz for 500 cycles/day)	The external stimulation applied in this study did not significantly enhance the fracture healing process. However, 0.5 mm/10 Hz stimulation induced the highest periosteal callus area.	Delay (7 days)^252^
Sheep model with diaphyseal osteotomy (24 subjects). Axial movement: 1.5 mm displacement with preloaded spring with a force of 40 N; Shear movement: allowing 1.5 mm sliding between distal and proximal bone segment, with 2% rotational slackness.	Shear movement considerably delayed the fracture healing, with only 60% bridging of osteotomy fragments in the Shear group, whereas 100% in the Axial group. Peripheral callus formation in the Shear group also reduced to 64% compared to the Axial group.	Immediate^253^
Rabbit fix-sliding model with diaphyseal osteotomy (64 subjects). Rabbits in the Axial and Shear group were subjected to self-body-weight induced compression, with different directions restrained by the fix-sliding devices.	The shear movement led to superior healing 4 weeks after fracture but inferior outcomes 2 weeks after fracture compared to axial interfragmentary movement.	Immediate^255^
Mouse model with tibia osteotomy followed by intramedullary nailing (80 subjects). Cyclic compression loading (0.5, 1 or 2 N; 1 Hz for 100 cycles/day, 5 days/week for 2 weeks)	Compared to the immediate loading model, the low magnitude (0.5 N, 1 Hz for 100 cycles/day, 5 days/week for 2 weeks) axial cyclic compression with a short delay (4 days delay) significantly improved fracture healing with increased callus strength. Such improvement diminished with increased loading amplitudes (0.5–2 N).	Immediate and delayed (4 days)^258^
Mouse model with femur osteotomy (20 subjects). Cyclic strain (8–16 N, 10 Hz, 3000 cycles, 3 times/week for 4 weeks)	Cyclic strain applied on the mouse fracture model led to significantly higher callus formation and mineralization within the remodeling phase, which is associated with Wnt signaling activation and reduced distribution of sclerostin and RANKL in fracture callus.	Delayed (3 weeks)^259^
Human tibia bone surrogates model with transverse osteotomy*. Dynamic compression loading (150/200 N^−1^ Hz for 5 h)	Dynamic loading increased the transport of bone cells (280% for chondrocytes and 180% for osteoblasts) and growth factors (220% for chondrogenic growth factors and 120% for osteogenic growth factors) in the callus compared to the free diffusion. A moderate transport improvement was observed for the MSCs (22%) and fibroblasts (17%).	Simulated loading^260^
Sheep metatarsus fracture model*. Cyclic compression loading (0.02 mm displacement of amplitude; 1, 50, and 100 Hz for 15 min)	Mechanical loading with low amplitude and high frequency (0.02 mm displacement, 50 and 100 Hz) significantly improved the osteogenic activity of the callus. Interstitial fluid flow velocity was the only mechanical variable undergoing a significant increase in amplitude and peak value when the frequency of the external stimulus increased.	Simulated loading^261^
Sheep fracture model*. HF/LM Vibration (1% displacement, 90 Hz for 5 min, 2 times/day)	HF/LM-mediated improvement of bone formation could attribute to an increase in the interstitial fluid flow velocity, which promotes endochondral ossification, cell proliferation, migration, and ECM synthesis	Simulated loading^273^
*Defect healing, bone loss regeneration, and implant healing*		
Mouse model with critical-sized tibia defect. The defective limb was subjected to daily loading of 5 N peak load, 2 Hz, 60 cycles for 4 consecutive days.	Loading during the inflammatory phase (post-surgery day, PSD 2–5) delayed hematoma clearance and bone matrix deposition and stimulated cellular proliferation and osteoclast activity. Loading during the matrix deposition phase (PSD 5–8) stimulated cellular proliferation and promoted cartilage and bone formation. Finally, loading during the remodeling phase (PSD 10–13) stimulated cellular proliferation and prolonged the remodeling phase.	Delayed (1 day)^256^
Rat model (156 subjects) with anterior cruciate ligament reconstruction. Cyclic axial loading (0.05 Hz, 30 g with 2.2% graft elongation tensile).	Delayed application of cyclic axial loading after anterior cruciate ligament reconstruction resulted in improved mechanical and biological parameters of tendon-to-bone healing, manifested by improved bone formation, less ED1^+^ inflammatory macrophages, more ED2^+^ resident macrophages, fewer osteoclasts, and reduced tissue vascularity.	Immediate, early delayed (4 days), and late delayed (10 days)^257^
Healthy rat ulna model (29 subjects). Static axial compression (8.5 and 17 N, 10 min/day for day 1–5 and 8–12) and axial dynamic compression (17 N, 2 Hz, 1200 cycles/day for day 1–5 and 8–12)	Static loading could not generate an anabolic bone response, whereas it suppresses skeleton growth. Dynamic loading significantly promotes periosteal and endocortical bone formation compared with static loading.	Immediate^266^
Healthy rat ulna model (29). Axial dynamic compression (haversine waveform at 17 N peak value, 360 ×1 cycles/day or 90 ×4 cycles/day, 3 days/week for 16 weeks)	The loaded ulnas showed 5.4% (360 ×1) and 8.6% (90 ×4) greater areal bone mineral density than the control. In addition, bone mineral content was enhanced by 6.9% and 11.7% in the 360 × 1 and 90 ×4 loaded ulnas.	Immediate.^269^
Mouse model with type I diabetes-induced bone loss (133 subjects). Cyclic axial compression (1.2–2.4 N, 2 Hz, 120 cycles/day for 3 days)	The combination of mechanical loading and PTHrP-derived peptide overcame the bone loss, fragility, and reduced mechanoresponsiveness caused by diabetes.	Immediate^262^
OVX Mouse model with undamaged bone (150 subjects). Compression loading (4 N, 10 Hz, 5 min/d for 2 weeks) was applied on the lumbar spine in the dorsal-ventral direction.	The loaded OVX mice showed a significant increase in the number of osteoblasts and a decrease in the number of osteoclasts via Wnt3a signaling. Spinal loading also elevated the volume of microvascular and VEGF levels.	Immediate (2 weeks after OVX surgery)^263^
Rat tibia model with titanium implant placement (77 subjects). Compression loading: HF/LM (40 Hz, 0.5 N); HF/HM (40 Hz, 1 N); LF/LM (2 Hz, 10 N); and LF/HM (2 Hz, 20 N).	Bone fraction and bone-implant-contact rate were increased at the cortical level in response to HF/LM and LF/HM loading. However, BIC at the medullar level was positively influenced only in response to HF-LM loading	Immediate^272^
Rabbit tibia model with titanium implants (4 implants/rabbit) placement (10 subjects). Movement loading (20 min daily treadmill running from 0 to 6 weeks)	The test group showed more significant vertical bone growth (1.26 ± 0.48vs 0.32 ± 0.47 mm, *p* < 0.001), higher ISQ values (11.25 ± 6.10vs 5.80 ± 5.97 *p* = 0.006), higher BIC (25.14 ± 5.24%vs 18.87 ± 4.45%), and higher bone neoformation (280.50 ± 125.40 mm^2^ vs. 228.00 ± 141.40 mm^2^, *p* = 0.121).	Immediate (with 2 weeks progressive adaption phase)^271^

* Finite elemental analysis and mathematical modeling.

### Mechanoresponses of bone regeneration in vitro

**Figure 5.**
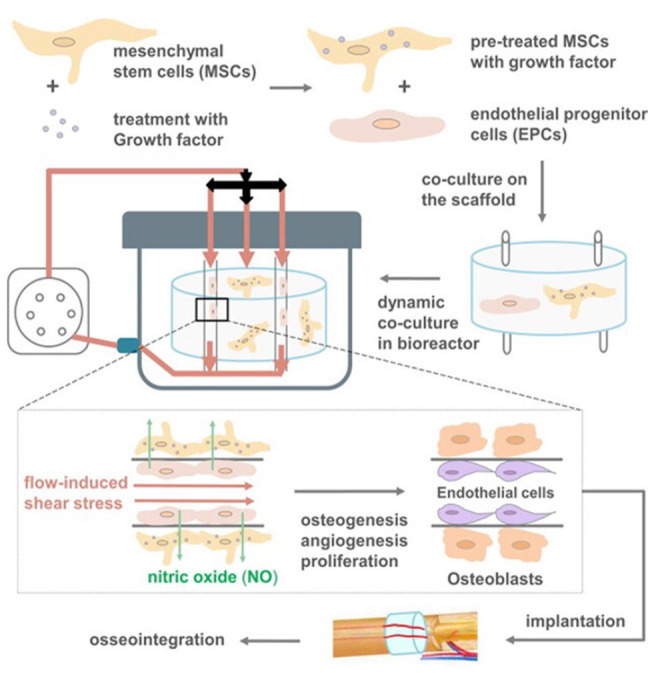
The role of the bioreactor in vascular network progression. Figure reused with permission from Mokhtari-Jafari et al.^289^ according to license agreement 5271260413964. © 2020 Elsevier Ltd. All rights reserved.

Mechanical load is a type of physical signaling that can impact the host cell functions, including proliferation, migration, matrix orientation, and enzyme secretion.^277^ Applying external mechanical stimulation to bone tissue engineering (BTE) could improve bone tissue development.^278,279^ Bioreactors have been reported to provide mechanical stimuli, such as fluid flow, to allow nutrient migration to cells, thereby increasing cell viability and promoting bone regeneration (Figure 5).^267,268,280^ Different bioreactors (such as rotating wall vessel reactor, pinner flask, and flow perfusion) have been introduced for applying mechanical stimuli.^267,268^ A previous study compared the impact of static and bioreactor cultures on scaffolds-based polycaprolactone-tricalcium phosphate (PCL-TCP) seeded with human fetal mesenchymal stem cells (hfMSC). Compared to the static culture environment, the biaxial rotating bioreactor considerably enhanced the osteogenic differentiation and proliferation of hfMSC.^267^ Liu et al. provided a 3D-culture system for human bone mesenchymal stromal cells (hBMSC) encapsulated in a scaffold-based polyurethane. The results indicated that both differentiation and proliferation of hBMSC were improved under the exertion of on-off cyclic mechanical compressions (10% strain) and perfusion (10 ml/min) for about 2 weeks of culture.^281^ Another study reported that cyclic mechanical stimuli improved the osteogenic differentiation of MSCs in demineralized bone scaffolds.^282^ Ignatius et al. investigated the effects of cyclic strain on human fetal osteoblasts (hFOB 1. 19). The results showed that uniaxial mechanical strain (1%, 1 Hz, 1800 cycles/day for 3 weeks) promoted the proliferation, differentiation, and osteogenic gene expression on osteoblasts.^283^ In addition, continuous compression (0–10.0 g/cm^2^ for 48 h) stimulated the OPG production of mouse osteoblasts (MC3T3-E1) via a non-canonical Wnt/Ca^2+^ pathway. The enriched OPG inhibited osteoclastogenesis by blocking the RANK/RANKL interaction.^278^ van Eijk et al. also evaluated the effect of the timing of mechanical stimuli on the proliferation and differentiation of BMSCs cultured on braided poly (lactic-co-glycolic acid) (PLGA) scaffolds. The application of loading during cell seeding appears to be influential in the differentiation and proliferation as opposed to applying loading immediately after cell seeding or with a delay.^284^ A previous study reviewed the impact of mechanical loading on hMSCs and focused on BTE challenges.^285^ This study reviewed four types of mechanical loads, including compression, perfusion, vibration, and stretching. Various mechanical loadings can induce osteogenesis in hMSCs via different or similar metabolic routes. For instance, dynamic compression might activate calcium signaling that upregulates the phosphorylation of ERK1/2, therefore, enhancing the FOSB expression in hMSCs.^286^ C-Jun protein and FOSB protein then can form a complex known activator protein-1 (AP-1) that readily links to DNA, causing an enhancement in the transcription rates of *RUNX2* and other osteogenic genes.^285^ In addition to the conventional osteogenic functions, mechanical loading also leads to epigenetic changes in bone cells, which is central to cellular differentiation and stem cell lineage commitment. FSS (rocking platform at 0.5 Hz, 1.5 cm amplitude for 24 h) was reported to suppress DNA methylation for late-stage osteogenic markers (OPN) in mouse osteocytes (MLO-Y4) and MSCs, increasing gene availability for expression.^287,288^

Several recent studies have reported the improved osteogenic functions of osteoblasts and MSCs in response to mechanical loading (Table 2). These studies based on stretching or compression models have non-negligible limits. As mentioned above, bone tissue deformation during everyday locomotion and physical exercises ranges from 0.04% to 0.3%, with a rare occurrence exceeding 0.1%.^99^ In vitro studies reveal that the deformation required for bone cells to react to mechanical stimulation is significantly higher, ranging from 1% to 10%, which is 10 to 100 times greater than that needed for bone tissue, which would result in a fracture when stimulating bone cells in natural bone.^100,101^ Therefore, the deformation model with high magnitude may not be ideal for studying the mechanoresponsiveness of bone cells. Instead, the effects of FFS or low-magnitude deformation on osteocytes could be a potential and promising point to help us understand the mechanism of bone mechanoresponsiveness. As a bony mechanosensor, dendritic osteocytes and lacunar-canalicular systems work together to perceive and amplify subtle deformation or FSS generated by mechanical loading.^102,107^ This mechanism allows osteocytes to sense micro deformation generated by macroscopic and physiological mechanical loading.^101^ Furthermore, given the central regulatory role of osteocytes on osteogenic and osteoclastic functions,^57–61^ functional changes of osteocytes induced by FSS or deformation may be an ideal model to study bone remodeling in response to mechanical loading. However, how to translate macroscopic mechanical loading into deformation and fluid shear stress received by osteocytes remains unclear. Biosensors with high sensitivity are needed to quantify the pressure and deformation inside the bone matrix. At the same time, it is imperative to establish a unified mathematical model platform based on different mechanical loading parameters and animal models (species, location, and physical properties of bone). Using such platforms, researchers can calculate the loading parameters generated by physiological movement and fierce exercises at the cellular level. Such parameters facilitate establishing an in vitro model of the effect of “real world” mechanical loading on bone cells, which is of great significance for elucidating the mechanism of bone mechanoresponsiveness and establishing a therapeutic mechanical loading strategy that promotes anabolic bone remodeling.

**Table 2.** The summary of recent in vitro studies on the impacts of three types of mechanical loading on bone cells.^290^.

Amount of load/Type of system used	Result	Publish year	Reference
Compressive Pressure*			
490.33 Pa (5.0 g/cm^2^)	No considerable impact was found on the viability of MC3T3-E1 cells	2017	Shen et al.^291^
196.13–392.26 Pa (2.0 g/cm^2^–4.0 g/cm^2^)	The differentiation of osteoblast was increased with increasing compressive stress	2013	Tripuwabhrut et al.^292^
98.07 Pa (1.0 g/cm^2^)	It is considered an optimal parameter for the osteoblastic differentiation	2008	Yanagisawa et al.^293^
*Tensile Strain* ^&^			
Cyclic stretching (5% strain at a frequency of 1.0 Hz for up to 12 h by the FX-4000 Flexcell) was exerted on macrophages. The stretched macrophages have been co-cultured with BMSCs	The expression of RUNX2 and OPN was considerably increased with induced YAP activation and nuclear translocation, which later manipulated the expression of downstream BMP2 to promote BMSCs osteogenesis	2021	Dong et al.^294^
10% uniaxial static strain was exerted on osteoblasts by a homemade multiunit cell stretching and compressing device	The osteoblasts’ proliferation and the expression levels of mRNA and protein of alkaline phosphatase (ALP), osteocalcin (OCN), Runt-related transcription factor 2 (Runx2), collagen type I, hypoxia-inducible factor 1-alpha (HIF-1*α*), and vascular endothelial growth factor (VEGF) were considerably more than in the non-stretch control categories	2020	Li et al.^42^
Flexcell tension system in vitro for applying mechanical stretching of human jaw bone marrow MSCs	Considerably increased calcium deposition and ALP activity. The expression levels of Runx2 and osterix were markedly upregulated, while NF- kB was downregulated	2018	Chen et al.^295^
*Fluid Shear Stress* ^#^			
1.2 Pa (12 dyn/cm^2^) Fluid shear stress for 30, 60, and 90 min was applied to MC3T3-E1 cells	The expression level of long-coding (RNA) lncRNA taurine upregulated 1 (TUG1) enhanced in a time-dependent behavior. LncRNA TUG1 upregulated expression of fibroblast growth factor receptor 1 (FGFR1) by sponging miR34a, which promoted the proliferation of osteoblast and prevented osteoblast apoptosis	2021	Wang et al.^296^
Laminar flow 1.2 Pa (12 dyn/cm^2^) was applied on the MC3T3-E1 cells for 1 h after incubation in serum-free medium for 6 h	Downregulated miR-140–5p and promoted the proliferation of osteoblast by activating the VEGFA/ERK5 signaling pathway	2021	Wang et al.^297^
1.2 Pa (12 dyn/cm^2^)	Inducing proliferation of MG-63 cells and up-regulated the expression level of focal adhesion kinase (FAK)^%^	2021	Lei et al.^298^
The mouse osteoblast cell line, MC3T3-E1, was cultured in α-minimum essential medium (α-MEM). Fluid shear stress was applied to cells by placing T25 flasks or 6-well culture dishes on a horizontal shaking apparatus fixed in a culture incubator and shaken at 100–120 rpm	Fluid shear stress increased the Piezo1 in MC3T3-E1 cells’ expression, activated the AKT- serine-threonine protein kinase glycogen synthase kinase 3 (GSK3)/β-catenin pathway and regulated the expression level of RUNX-2	2020	Song et al.^299^
Mouse osteoblasts (MC3T3-E1) were exposed to the pulsating fluid flow (peak shear stress rate: 6.5 Pa/s, amplitude: 1.0 Pa, frequency: 1 Hz) for 1 h	FSS induced changes in the arrangement of F-actin in one direction, causing them to be uniform and more compact, and enhanced the expression level of phospho-paxillin and integrin α5	2020	Jin et al.^300^
FSS at 2 Pa (20 dyn/cm^2^)	Inducing a 126% enhancement in hMSC proliferation over static controls	2006	Riddle et al.^301^
Mouse osteocytes (MLO-Y4) on exposed to FSS (rocking platform) at 0.5 Hz with an amplitude of 1.5 cm for 24 h	With FSS stimulation, the DNA methylation decreased for osteogenic markers, facilitating the availability of genes for expression.	2015	Chen et al.^287^

* The results showed that continuous compression positively impacts the differentiation of MC3T3-E1 cells and osteoblasts. However, the exact dose of continuous compression for MC3T3-E1 cells and specific impacts are currently unclear.^290^

& Different kinds of stress can create relative strain when exerted on an object. The ratio of the shape, length, and volume of the object’s variation before and after the action of tensile stress (single/bidirectional tensile stress) is known as tensile strain.^302^

# Fluid shear stress is mechanical stress from the extracellular fluid, like tissue fluid, flowing in the cell membrane surface.^303^ Applying loads (including muscle contractions, blood pressure, mechanical loads, and lymphatic drainage) to the bones leads the interstitial fluid to flow, compressing the lacunar-canalicular system and inducing different mechanical stimuli like fluid shear stress.^100,111,304^

% FAK may play an essential role in the mechanical leading of the implant-bone interface.

## The effect of mechanical loads on vascularization

### Why vascularization and angiogenesis are important?

One of the major challenges in bone tissue engineering (BTE) is achieving successful and sufficient vascularization after implantation, which is crucial for providing the necessary nutrients to support cell growth within the scaffolds.^305^ Host tissue can provide blood vessels and nutrients to aid healing after implanting scaffolds. In addition, biomaterials and composites can create an environment that facilitates the release of specific biochemical cues after implantation, which targets wound healing. These signals initiate blood vessel ingrowth. Consequently, more blood vessels are directed into the injured tissue.^8^

Blood vessels are produced through two biological processes, which are crucial to osteoporosis. Hemangioblasts are mesodermal cells that migrate to a specific location during early development and assemble to create the primary vessels in angiogenesis.^306^ Most of the newly formed blood vessels sprout through angiogenesis, accompanied by the growth of the current vascular networks through several processes such as endothelial cell migration, sprouting vessel pruning, and anastomosis.^307,308^ Endochondral and intramembranous ossification are separate processes through which bones are formed. Osteoblasts that can form bone must be present, and bone growth must be accompanied by neovascularization. Angiogenesis-osteogenesis coupling describes how bone production happens in a spatial and temporal link with the vascularization of the ossifying tissue.^309–311^ Throughout the angiogenesis process, endothelial cells (ECs) develop, migrate, form tubes, and eventually create conduits where blood flows and supplies the essential nutrients, growth factors, hormones, and oxygen for the bone cells. Also, blood vessels deliver the hematopoietic precursors of osteoclasts to the site of bone resorption and cartilage to eliminate the consequences of ECM degradation. Additionally, the subendothelial walls of vessels include pericytes, which seem crucial in the linkage between osteogenesis and angiogenesis.^312,313^ Blood vessel formation in osteoporosis occurs through two critical biological processes. Hemangioblasts, mesodermal cells, assemble at specific locations during early development and create primary vessels in angiogenesis. Most blood vessels are formed through angiogenesis, which involves the growth of current vascular networks through endothelial cell migration, sprouting, vessel pruning, and anastomosis. Bones are formed through endochondral and intramembranous ossification, which require the presence of osteoblasts and neovascularization. The coupling of angiogenesis and osteogenesis describes the spatial and temporal link between bone formation and vascularization of the ossifying tissue. Throughout angiogenesis, endothelial cells develop, migrate, form tubes, and create conduits for blood flow, delivering essential nutrients, growth factors, hormones, and oxygen to bone cells. Additionally, blood vessels transport hematopoietic precursors of osteoclasts to sites of bone resorption and cartilage to remove the consequences of extracellular matrix degradation. The subendothelial walls of vessels contain pericytes, which play a crucial role in the linkage between osteogenesis and angiogenesis.

Angiogenesis is necessary for bone growth and development, bone health maintenance, and post-fracture repair.^314,315^ For instance, a previous study discovered that the blood supply in individuals with osteopenia or osteoporosis is significantly lower than that in individuals with healthy bone mass, demonstrating a strong correlation between bone density and blood supply.^316,317^ Moreover, it has been reported that endothelial Notch signaling encourages osteogenesis and angiogenesis in the bone microenvironment. It was demonstrated by the presence of 5-ethynyl-2′-deoxyuridine (EdU) labeled vascular ECs in the region where long bones in mice grew rapidly.^318^ Hence, promoting angiogenesis and vascularization can benefit bone formation/remodeling. The vasculogenesis stage is completed with primary vascular plexus formation. All transformations of the vascular net proceed within angiogenesis when new vessels are created from existing ones. At the angiogenesis stage, the initial vascular plexus considerably expands through capillary branching and changes into a highly organized vascular network.^319^ Angiogenesis starts from the local elimination of the wall of the pre-existing blood vessel as well as the activation of ECs proliferation and migration. ECs are recruited in tubular structures around which the blood vessel walls are created. During further maturation of the vascular network, capillaries fuse into larger vessels, veins, and arteries.^319^ The capillaries walls and fine vessels include a single layer of cells (pericytes), while walls of arteries and veins are formed by various smooth muscle cell layers.^320^ There are two key cell types in vessels: mural cells and endothelial cells. Therefore, it is prominent to understand the mechanism of angiogenesis to characterize which processes regulate the bioactivity of these cells and their interaction.^319^ In flat bones, bone thickness affects microvasculature’s patterning significantly. Thinner regions (less than 0.4 mm) possess only dural networks and periosteal, with larger vessels connecting the two sides of bone, lacking an actual vascular network.^321^ However, thicker and flat bones contain a microvascular network similar to long bones.^321,322^ Blood vessels of various regions in bone include distinct structures.^322^ Due to the close relationship between osteogenesis and angiogenesis, angiogenic growth factors are involved in endochondral ossification and neovascularization, making them prominent therapeutic targets for bone regeneration. For example, VEGF, a key angiogenic growth factor associated with bone healing, has a pivotal role in bone repair by promoting angiogenesis and stimulating significant skeletal cell populations, osteoblasts, chondrocytes, and osteoclasts.^323,324^

### How mechanical loading regulates vascularization

Previous research has demonstrated that mechanical loading increased osteogenic and angiogenic responses in bone. Matsuzaki et al. reported that skeletal fatigue cyclic compression (18.7 N, 2 Hz, 1650–5287 cycles) increased periosteal vascularity and regional bone area, with the coordination of angiogenesis and osteogenesis.^325^ Additionally, mechanical loading applies compressive forces to specific bone areas, allowing interstitial bone fluid to migrate from a high fluid-pressure area to a low fluid-pressure area, which promotes osteogenesis and inhibits the development of osteoclasts.^326^ The increased intramedullary fluid pressure enhances transcortical fluid flow, generating fluid shear stresses on bone cells and activating mechanoresponses. Since lower extremities exercises (done while standing upright) were more influential in bone mass augmenting than the same exercise in done-supine,^327^ it is supposed that gradients of fluid pressure affect bone remodeling.^327^ Frangos et al. reported that enhanced vascularization was associated with the increased interstitial fluid flow because of the leaky nature of capillaries.^329^,^331^ Vascularization can change osteoblastic functions by releasing endothelial-derived factors like endothelin and NO. Endothelin enhances DNA synthesis and inhibits alkaline phosphatase activity in osteoblasts, which may reflect an enhancement in the proliferation of osteoblasts.^328,329^ Endothelin also inhibits bone resorption and osteoclast margin ruffling (Q effect).^329,330^ On the other hand, NO released from endothelial cells also suppresses bone resorption by disturbing the osteoclast spreading. On exposure to NO, osteoclasts undergo significant retraction without the action of margin ruffling.^329,330^ Notably, the release of NO and endothelin in cultured ECs can be regulated by FSS via protein-kinase-C (PKC) and cGMP pathways.^329,331^ Another case study also found that delayed mechanical loading (body weight loading by daily activity, lasting 3 weeks, 4 weeks delay post-trauma) promoted critical-sized (8 mm displacement) fracture healing (20% more bone formation) and stimulated vascular remodeling in a rat model by increasing the number of large vessels while decreasing the number of small vessels. Whereas early mechanical loading inhibited vascular invasion into the defect (66% less) and reduced bone formation (75% less) compared to the non-loading control.^332^ Therefore, Vascular network remodeling and its coupling effect on bone regeneration are highly time-dependent in response to mechanical loading.^332^ Mechanical loads can affect some factors, including nephronectin (NPNT), VEGF, HIF-1, epidermal growth factor-like domain (EGFL), and Notch ligands. Such factors can regulate the differentiation and proliferation of ECs, encourage bone vascularization, and improve angiogenic and osteogenic coupling in the local bone microenvironment.^333,334^ Some investigations focused on how mechanical loading affects ECs and their angiogenic ability in vitro. It has been demonstrated that specific forces, such as hemodynamics forces (shear stress and cyclic strain generated by the blood flow), manipulate the commencement and development of angiogenesis, along with the function of ECs.^277,280,335^ For instance, Li and Sumpio reported that the proliferation of bovine aortic endothelial cells (BAECs) was enhanced by cyclic strain (10% strain, 1 Hz for ⩽24 h).^280^ Another investigation showed an enhancement in the migration (1.83 ± 0.1 folds) and tube formation of BAECs in response to cyclic strain (5% strain, 1 Hz for 24 h). Such enhancement can be weakened or abolished with the treatment of Pertussis toxin (a Gi-protein inhibitor), cRGD peptide (an integrin blocker), and siRNA silencing of MMP9 and urokinase-type plasminogen activator (uPA).^335^ Furthermore, Iba and Sumpio observed that cyclic strain regulated the ECs elongation by reorganizing the actin filaments network.^336^ A comparative study reported that the type of mechanical loading determined the response of mechanoreceptors in BAECs. In this study, ERK1 and ERK2 were activated 2- and 1.6-fold at 30 min by cyclic strain, whereas they were activated 11.7- and 14.4-fold at 5 min by FSS. FSS leads to more robust and rapid activation of ERK and p38 compared with cyclic strain.^337^

Although previous studies have clarified the positive effects of mechanical loading on osteogenesis and angiogenesis, few studies have focused on the mutual regulation and crosstalk of osteogenesis and angiogenesis under mechanical loading. Cheung et al. reported that mouse osteocytes (MLO-Y4) exposed to the physiologic fluid flow (1.0 Pa) were preserved from TNF-α mediated apoptosis.^338^ However, the absence of fluid flow led to the prevalence of osteocyte apoptosis, resulting in the release of VEGF.^338,339^ Apoptotic bone cells fail to inhibit osteoclast activation,^58,59,61^ resulting in the colocalization of bone resorption and angiogenesis promoted by VEGF.^338^ This study clarifies the relationship between inflammatory environment-mediated bone resorption and vascularization and provides substantial experimental evidence for mechanical loading against bone resorption but with certain shortcomings. TNFα may directly affect VEGF release, based on cell line and phenotype,^340,341^ and other inducers of apoptosis should be tested (especially mediators associated with unloading-associated bone resorption). Mechanical compression loading can activate human umbilical vein endothelial cells (HUVECs) on the demineralized bone scaffold embedded with alginate microspheres through increased VEGF release. VEGF and mechanical loading synergically activate HUVECs with elevated expression of MMP-2/9 and Flk-1 (a VEGF receptor and cytoskeletal component participating in mechanotransduction), thereby improving angiogenesis in vivo.^15^ Claes and Meyers hypothesized the relationship between interfragmentary movement direction and vascularization in fracture healing. Cycle compressive strain resulted in more vessel formation than the shearing or tensile strain.^342^

Although mechanical loading has been proven to improve angiogenesis, how to make the vascular network structure more conducive to bone formation through mechanical loading is still inconclusive. Certainly, the overabundance of the capillary network impairs bone formation. In contrast, the hierarchical structure of large blood vessels-small blood vessels-capillaries closer to healthy tissues is more beneficial for nutrient transport and waste exchange. In addition, due to the elasticity of blood vessels, whether the vascular deformation generated by blood flow can be employed to activate the mechanoresponsiveness of bone cells inside the bone defects deserves further study.

## Conclusions

Mechanical stimulation is essential for bone regeneration as it affects the biological functions of bone cells and endothelial cells. This review provided an overview of the basic structure of bone and the biological structures and functions related to mechanical loading, focusing mainly on the regulatory effects of mechanical loading on bone fracture/regeneration. The mechanosensation and mechanotransduction-related molecular structures and signaling pathways were analyzed in detail. Current in vitro and in vivo models used for research on the effects of mechanical loading on bone healing/regeneration were collected and compared. In addition, we briefly reviewed the role of mechanical loading in angiogenesis during tissue healing. Overall, the hierarchical structure of bone matrix can convert mechanical loading into deformation or fluid shear with different magnitudes and types, stimulating bone cells (osteocytes, as the primary bone remodeling regulator) and regulating following bone remodeling via skewing the balance of osteogenic/osteolytic functions. The signaling pathways related to mechanotransduction do not act independently but synergistically with the overall health status, hormone levels, and cytokine patterns in local tissue. Therefore, enhancing the activity of target molecules related to mechanosensation and mechanotransduction (such as integrins, FAK, Cx43, TAP/TAZ., etc.) may improve or restore the mechanosensitivity of bone cells under specific conditions, leading to ideal bone gain/maintenance.

In the study of mechanical loading-mediated bone fracture healing and regeneration, the molecular pathways and targets identified in vitro cannot be fully validated in animal models due to the unspecificity of cell lineage with conditional gene knockout in vivo. Moreover, the current in vivo and in vitro models are remarkably distinct, requiring an excellent mathematical model to convert the mechanical parameters between them. Only in this way can the parameters be standardized for future research, which also applies to the study of mechanical loading-mediated angiogenesis. Furthermore, the current research on osteogenesis and angiogenesis induced by mechanical loading is primarily based on coupled experiments. Therefore, from a logistical perspective, it is crucial to establish experimental models in which mechanical loading modulates osteogenesis through angiogenesis and vice versa, which helps to clarify the primary and secondary relationships between angiogenesis and osteogenesis.

In conclusion, although mechanical loading is considered a promising strategy for bone repair and regeneration, more studies are needed to elucidate their roles, limitations, shortcomings, and challenges for future research and application. The focus of research in this field is to match the models and parameters of in vivo and in vitro studies, screen out highly sensitive molecular targets that can improve the mechanoresponsiveness of bone cells, and form therapeutic strategies that are truly clinically applicable.
